# Broadband Electromagnetic Field Prediction at Unseen Wavelengths via Physics‐Guided Neural Operators

**DOI:** 10.1002/nap2.70242

**Published:** 2026-08-09

**Authors:** Joonhyuk Seo, Chanik Kang, Dongjin Seo, Haejun Chung

**Affiliations:** ^1^ University of California Irvine Irvine California USA; ^2^ Hanyang University Seoul South Korea; ^3^ Yale University New Haven Connecticut USA

**Keywords:** Maxwell's equations, metasurface, neural operators, surrogate solver

## Abstract

Surrogate solvers that deliver full‐wave accuracy across broad spectral ranges can unlock the rapid design and analysis of nanophotonic devices. We present a physics‐guided neural‐operator surrogate solver that predicts electromagnetic field distributions throughout the visible band from a permittivity map and a query wavelength, including wavelengths unseen during training. Our approach is built on two key ideas: (i) spectral consistency, which formalizes the intrinsic relationship between wavelength‐dependent field variations and spatial frequency, and (ii) a conditional embedding framework that comprises a refined wave prior. We validate the approach on three representative nanophotonic platforms—a single‐layer metasurface (metalenses), a five‐layer volumetric metasurface (spectrum‐splitters), and a freeform waveguide for photonic integrated circuits—achieving full‐wave fidelity while reducing mean field‐prediction error relative to prior operator‐learning baselines by up to 80.3% (single‐layer), 56.8% (multilayer), and 32.3% (waveguide). The solver remains compact (0.43 M parameters; 86.9% fewer than FNO) and accelerates simulation by ∼42× compared with a finite‐difference frequency‐domain solver while yielding robust broadband generalization. By providing continuous‐wavelength field predictions with full‐wave accuracy, this surrogate solver eliminates a key bottleneck in modeling and inverse design of metalenses, spectrum splitters, and emerging large‐area meta‐optics, offering a drop‐in engine for system‐level co‐optimization in nanophotonics.

## Graphical abstract

A physics‐guided neural operator is trained on electromagnetic field distributions at discrete wavelengths across diverse nanophotonic structures. At inference, given any permittivity map and query wavelength, the model predicts continuous broadband electric‐field distributions with full‐wave accuracy, including wavelengths unseen during training, reducing prediction error by up to 80% over prior baselines.

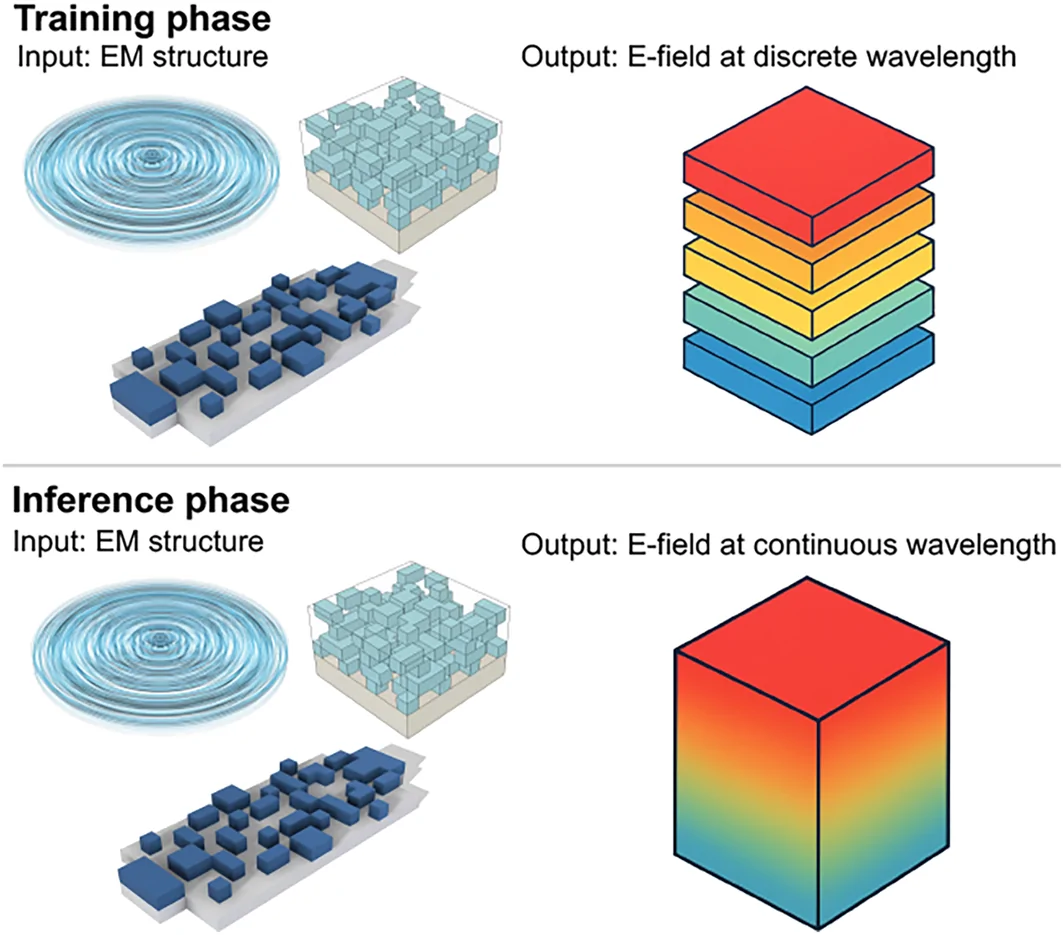

## 1 Introduction

Recent advances at the intersection of deep learning (DL) and photonics have led to significant breakthroughs across various domains, including the inverse design of photonic structures [1, 2, 3, 4, 5], high‐resolution imaging and computational microscopy [6, 7, 8], optical signal processing and communication systems [9, 10, 11], and the realization of optical neural networks [12, 13, 14]. Specifically, DL‐based approaches have demonstrated remarkable capabilities in rapidly predicting electromagnetic phenomena traditionally analyzed through numerical methods [2, 15, 16, 17]. By training neural networks on datasets derived from simulations or experiments, DL approaches achieve orders‐of‐magnitude acceleration in predicting electromagnetic fields, device performance, and structural optimizations, all while maintaining accuracy comparable to traditional numerical solvers [2, 15, 16, 17].

One important application of DL in photonics is the development of surrogate solvers that approximate electromagnetic simulations. These surrogate solvers predominantly utilize deep neural networks due to their rapid inference and notable learning capability. Traditional data‐driven neural network‐based surrogate solvers [15, 16, 18, 19, 20] learn end‐to‐end mappings from structure/excitation to fields or responses, approximating the input–output operator of Maxwell's equations. However, these approaches frequently encounter limitations in accurately capturing the underlying physical characteristics of Maxwell's equations [2].

To address these shortcomings, physics‐informed neural networks (PINNs) [21, 22, 23] and physics‐regularized learning methods [2, 24] have been widely explored. PINNs incorporate Maxwell's equations into the training objective by penalizing PDE residuals and boundary/source conditions, enabling training without labeled simulation data. Physics‐regularized methods, by contrast, augment supervised/data losses with physics‐based penalties, reducing but not eliminating the need for labeled data. Despite these advantages, standard PINNs are largely instance‐specific: unless the network is explicitly conditioned on simulation parameters (e.g., wavelength and relative permittivity $\epsilon_{r}$) and trained across their ranges, they typically require retraining for each parameter setting. Even with parameter conditioning, accuracy often degrades at parameter values not covered during training, leading to reduced field‐prediction fidelity on unseen simulation settings.

Recently, surrogate solvers utilizing neural operators have gained attention, as these methods effectively learn mappings between infinite‐dimensional function spaces, reflecting the intrinsic characteristics of partial differential equations (PDEs) [25, 26]. Notable progress in operator learning includes transformer‐based neural operators [27, 28, 29, 30], convolutional neural operators [31], and Fourier neural operators (FNO) [17, 26, 32, 33, 34, 35]. Among these, FNO has demonstrated strong inference performance across various electromagnetic applications [17, 36, 37], making it particularly promising for accelerating electromagnetic simulations.

Nevertheless, recent surrogate solver studies, including FNO‐based methods, exhibit limited prediction accuracy under the untrained simulation conditions, thereby failing to demonstrate the generalization capability of these models. The notable decline in predictive accuracy at intermediate wavelengths between discretely sampled training points raises fundamental concerns about whether these models adequately capture the underlying physics governing electromagnetic phenomena.

In this work, we present a surrogate solver that delivers highly accurate, full‐wave solutions of Maxwell's equations across a continuous, broadband spectrum, even when training data are available only at sparsely sampled wavelengths (Figure 1). Our model maintains robust accuracy on untrained intermediate wavelengths, overcoming the generalization limits of existing surrogate solvers. Moreover, by leveraging the intrinsic scale invariance of Maxwell's equations [38], our demonstrations satisfy the solutions of Maxwell's equations at any target frequency band by multiplying a scaling factor.

**FIGURE 1:**
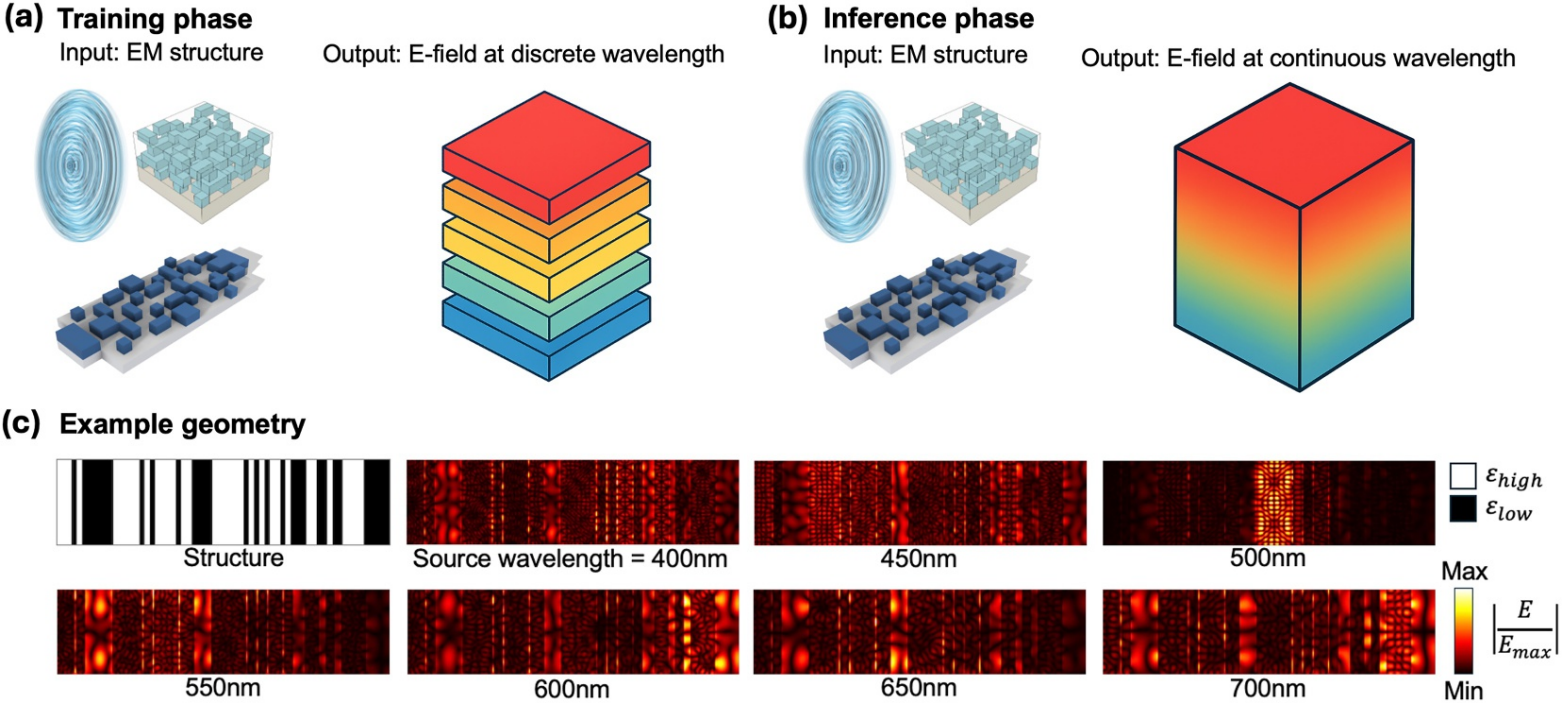
Overview of the approach. (a) *Training:* the network is supervised on paired permittivity profiles (maps) and electric‐field distributions over the simulation domain. Field data are provided at discrete wavelengths uniformly spaced every 20 nm across the visible spectrum. (b) *Inference:* given a permittivity profile and a query wavelength, the trained model predicts accurate full‐wave field distributions, including at unseen wavelengths. (c) *Example geometry:* schematic of a 2D subwavelength grating and its electric‐field distribution under normal‐incidence plane‐wave illumination.

## 2 Broadband Maxwell Surrogate Solver

Similar to a prior work [25], our model has an iterative process to map between infinite‐dimensional function spaces Figure 2.

(1) $$u=G_{\theta }\left(a\right)=\left(Q\;◦\;L^{\left(L\right)}\;◦\;\text{⋯}\;◦\;L^{\left(1\right)}\;◦\;P\right)\left(a\right)$$

where $L^{\left(l\right)}$ is a l‐th operator layer, L indicates the number of layers, and P, Q are lifting and projection operators, respectively. We define the layers $L^{\left(l\right)}$ as follows:

(2) $$L^{\left(l\right)}\left(z^{\left(l\right)}\right)=z^{\left(l\right)}+W_{2}^{\left(l\right)}\sigma \left(W_{1}^{\left(l\right)}{\tilde{K}}^{\left(l\right)}\left(z^{\left(l\right)}\right)+b_{1}^{\left(l\right)}\right)+b_{2}^{\left(l\right)}$$

where ${\tilde{K}}^{\left(l\right)}$ is our kernel integral operator based on the Fourier transform [32].

**FIGURE 2:**
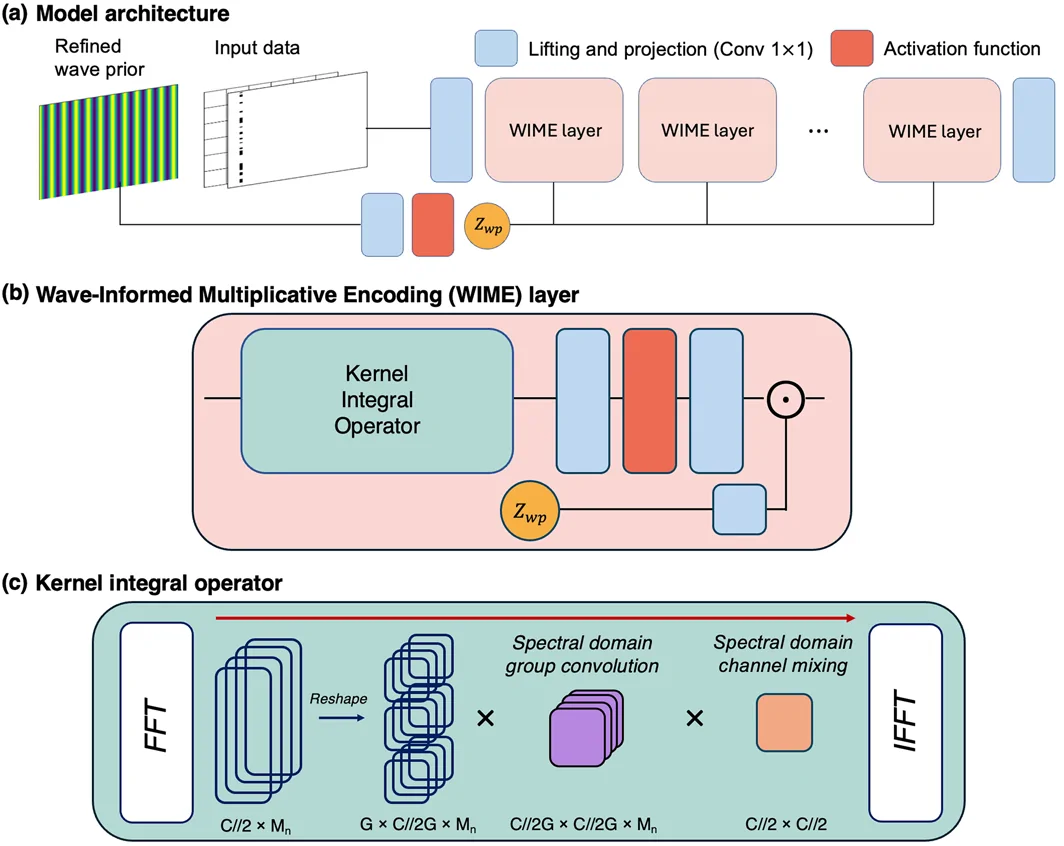
Model architecture and WIME layer. (a) *Network architecture:* the polarization‐dependent wave prior (conditional embedding) and the permittivity profile (input) are lifted with 1×1 convolutions (blue) and then passed through a stack of wave‐informed multiplicative encoding (WIME) layers (pink). The latent refined wave‐prior embedding $Z_{wp}$ is injected at every layer. (b) *Single WIME layer:* features are first updated by a kernel integral operator (green), followed by 1×1 convolutions (blue) and a nonlinear activation (red); the projected wave‐prior embedding modulates the feature map via element‐wise multiplication (⊙). (c) *Kernel integral operator schematic.*

Following prior work [33, 39], we employ a residual block consisting of two linear transformations separated by a nonlinear activation, which improves representational capacity and facilitates optimization. To preserve discretization invariance, we use pointwise 1×1 convolutions in place of spatial 3×3 convolutions, in contrast to ref. [17].

### 2.1 Wave Prior

As illustrated in Figure 3, the polarization‐dependent wave prior $W=\left[W_{x},W_{y}\right]$ is a synthetic wave pattern derived from homogeneous‐medium solutions of the wave equation. It is defined as

$$\begin{array}{ll} W_{x} & =\exp\;\left(i\;\frac{2\pi }{\lambda }\sqrt{\epsilon_{r}}\;x1^{\;⊤}\;\Delta \ell_{x}\right),\\ W_{y} & =\exp\;\left(i\;\frac{2\pi }{\lambda }\sqrt{\epsilon_{r}}\;1y^{\;⊤}\;\Delta \ell_{y}\right), \end{array}$$

where $\epsilon_{r}\in C^{H\times W}$ is the spatial map of relative permittivity, λ is the wavelength, $\Delta \ell_{x}$ and $\Delta \ell_{y}$ are the grid spacings, x and y denote the coordinate index vectors along x and y, and 1 is a vector of ones used to tile coordinates over the 2D grid. Because it originates from a homogeneous‐medium solution, W reproduces the wavelength‐dependent oscillation patterns observed in simulator‐generated fields within uniform regions.

**FIGURE 3:**
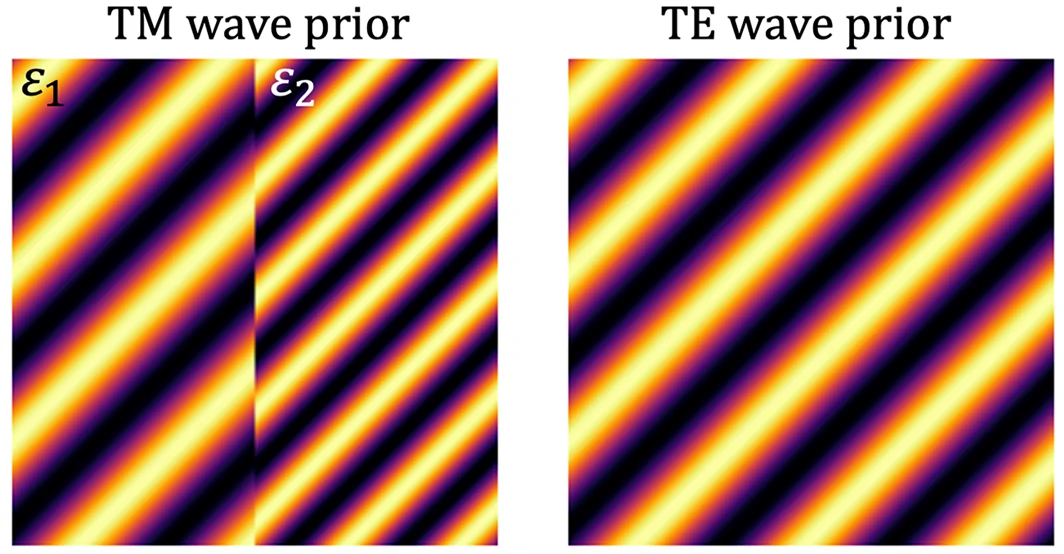
TM and TE wave priors. The TM wave prior $W_{x}=e^{j\frac{2\pi \sqrt{\varepsilon_{r}}}{\lambda }\;x1^{T}\;\Delta l_{x}}$ incorporates the spatially varying relative permittivity $\varepsilon_{r}$; the pronounced vertical seam marks interfaces where $\varepsilon_{r}$ changes and the normal electric field is discontinuous. In contrast, the TE wave prior ${\tilde{W}}_{x}=e^{j\frac{2\pi }{\lambda }\;x1^{T}\;\Delta l_{x}}$ omits the $\varepsilon_{r}$ factor, yielding a smooth, plane‐wave‐like pattern.

At material interfaces, the fields satisfy electromagnetic boundary conditions. For transverse‐electric (TE) polarization, the tangential electric field is continuous, $E_{t,1}=E_{t,2}$. For transverse‐magnetic (TM) polarization, the normal component of the electric displacement is continuous, $\epsilon_{1}E_{n,1}=\epsilon_{2}E_{n,2}$, where $\epsilon_{1}$ and $\epsilon_{2}$ are the relative permittivities of the adjoining media.

However, the wave prior used in this work is derived solely from the macroscopic permittivity map. It thus does not account for other complex electromagnetic phenomena in sub‐wavelength structures, such as wavelength‐dependent resonances and evanescent fields. Consequently, it exhibits sharp and physically implausible features in regions where the permittivity changes.

### 2.2 Spectral Consistency

As discussed earlier, complex optical effects such as scattering and resonance induce nonlinear, wavelength‐dependent variations in electric field profiles, which can impede accurate predictions at wavelengths not encountered during training. To address this challenge, the model must encode the relevant physics at arbitrary wavelengths, including unseen ones, by (i) constructing *conditional embeddings* that reliably capture wavelength‐dependent features and (ii) designing conditioning mechanisms that effectively integrate these embeddings throughout the network.

As illustrated in Figure 4, shorter wavelengths generally yield field patterns with higher spatial frequencies, whereas longer wavelengths produce sparser, lower‐frequency structures. This inverse relationship is inherent to solutions of Maxwell's equations and follows from ν=1/λ, where λ and ν denote the wavelength and spatial frequency, respectively [40]. We define *spectral consistency* as the requirement that wavelength‐dependent conditional embeddings reflect this inverse relation and that their spatial‐frequency content aligns with that of the desired electromagnetic‐field distributions. Embeddings that satisfy spectral consistency provide an informed prior on the expected field complexity at target wavelengths, including those absent from the training set, thereby facilitating robust broadband generalization in surrogate models.

**FIGURE 4:**
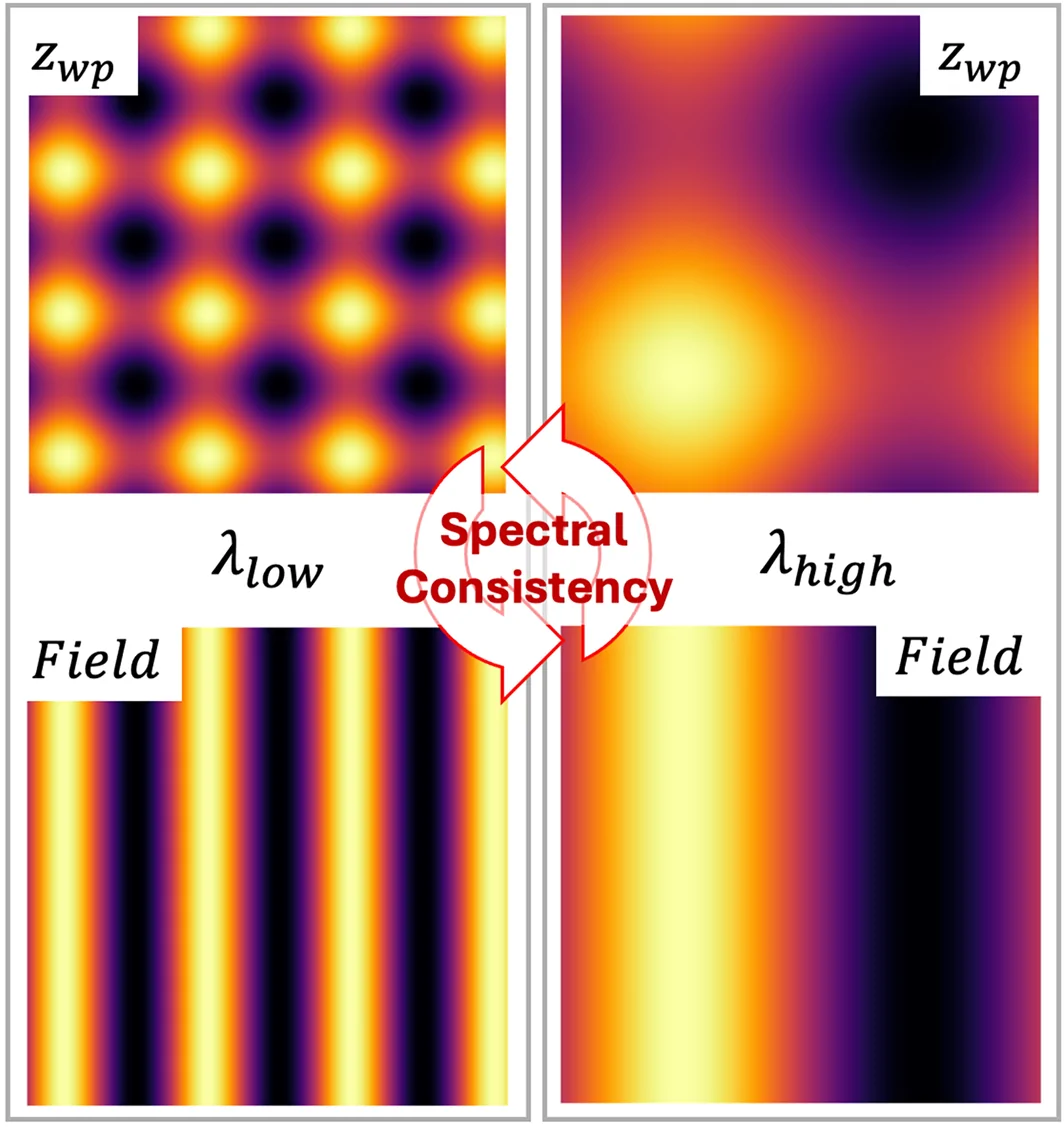
Spectral consistency. Across wavelengths, the latent wave prior (top) and the corresponding predicted electric‐field distributions (bottom) exhibit similar spatial‐frequency content, indicating that their spectra remain aligned under wavelength variation.

In this setting, conditional information that satisfies spectral consistency encodes the expected field complexity and spatial‐frequency distribution at target wavelengths, including those not seen during training. Enforcing spectral consistency, therefore, substantially enhances the surrogate model's broadband generalization.

## 3 Result and Discussion

In the following, we compare the field prediction performance for the continuous‐spectrum interpolation within the targeted bandwidth, including untrained intermediate wavelengths, in various simulation environments. We select three representative optical structures to investigate the generalization performance of our model under diverse electromagnetic phenomena.

### 3.1 Single Layer Metasurface: Metalens

Metasurfaces enable precise wavefront engineering by imposing arbitrary, spatially varying phases and amplitude profiles on transmitted or reflected waves. This versatility has been exploited in a wide range of applications, including beam steering [41, 42, 43], high‐resolution imaging [6, 7, 8], holography [44, 45, 46], vortex beam generation [47, 48], and communications [9, 10, 11]. Owing to their subwavelength thickness, metasurfaces provide a compact alternative to bulky optics and can be manufactured at scale using CMOS‐compatible lithographic processes [49].

Meta‐atoms in metasurfaces are individual scatterers and resonators that produce a prescribed macroscopic wavefront. Due to the resonance property of the meta‐atoms, the meta‐atoms project Maxwell's solutions nonlinearly over the varying wavelengths. This hinders large‐scale metasurface simulations; therefore, most of the metasurface studies assume a local periodic assumption for domain decomposition. This assumption often greatly degrades metasurface efficiency when the required phase and amplitude vary rapidly over the design region [50]. Therefore, it is very crucial to develop full‐scale metasurface simulators. Here, we apply our model to predict broadband solutions of Maxwell's equations in a single metasurface example, which can be applied to a metalens design.

Metasurface unit cells, often referred to as meta‐atoms, serve as subwavelength scatterers and resonators that collectively synthesize a prescribed macroscopic wavefront. Because each meta‐atom's resonant response varies nonlinearly with wavelength, simulating an entire metasurface at full scale becomes computationally expensive. To mitigate this, most designs invoke a local periodicity assumption for domain decomposition [51], but this approximation may not hold whenever the required phase and amplitude profiles change rapidly across the surface [50]. Such limitations impose the necessity for full‐scale metasurface simulators. Here, we apply our broadband surrogate model to predict full‐wave Maxwell solutions for a single‐layer metasurface [52, 53], illustrating its potential applicability to metalens design.

#### 3.1.1 Problem Setup

In this study, we utilize the open‐source FDFD simulation tool Ceviche [54] to generate electromagnetic field data for photonic structures. All simulations employ a uniform grid resolution of 40 points per micrometer and include a 1‐μm‐thick perfectly matched layer at the boundaries. For all cases, electric field distributions are generated under normally incident plane‐wave illumination for wavelengths spanning 400–700 nm. The single‐layer metalens is simulated over a 5.2μm×6.57μm domain using ${\text{SiO}}_{2}$ (refractive index 1.46) and air in 25 nm grid patches. Boundary conditions include a 1‐μm PML on all sides Figure 5.

**FIGURE 5:**
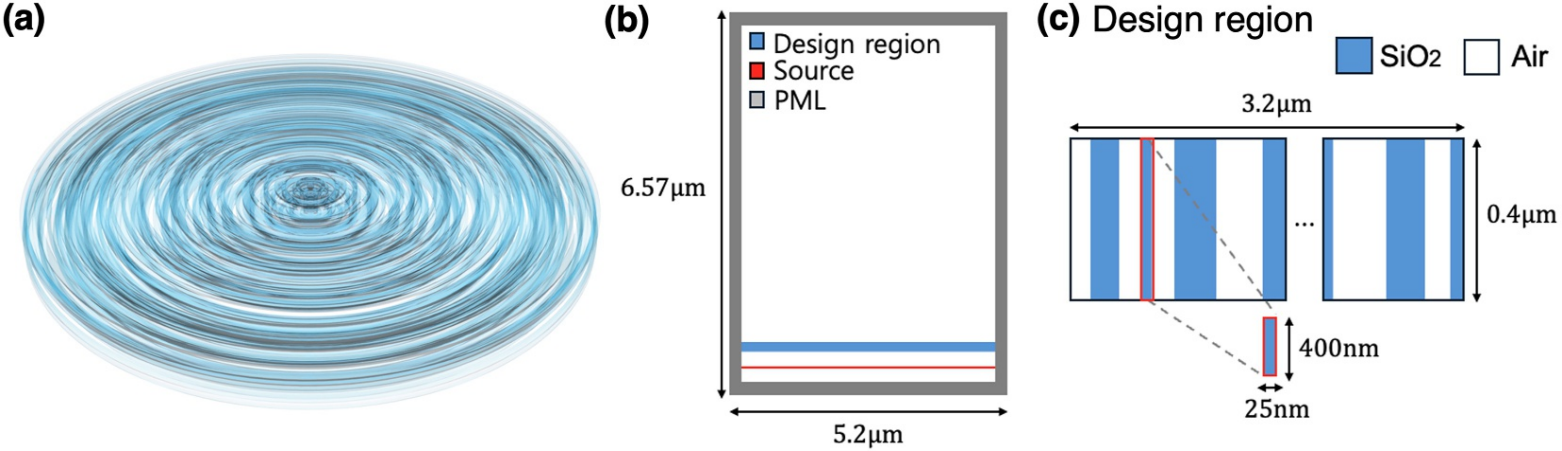
Single‐layer metasurface: geometry and simulation setup. (a) Schematic of the single‐layer metasurface. (b) Simulation setup for the single‐layer metasurface. (c) Detailed view of the design region within the simulation domain.

In addition, we compare the field prediction performance of UNet [55, 56], FNO2d [32], and F‐FNO2d [33], commonly used in surrogate solver study. Moreover, we evaluate our model against NeurOLight [17], the state‐of‐the‐art model for field prediction of a narrow wavelength range in electromagnetic simulations. For several baseline models (UNet, FNO2d, and F‐FNO2d), the conditional information was incorporated by simply concatenating the wave prior to the input. NeurOLight similarly utilized the wave prior concatenation, following the methodology proposed in the original study.

#### 3.1.2 Result

Figure 6 compares the proposed model's prediction with the full‐wave FDFD reference for the single‐layer metasurface and shows the corresponding absolute error. The left and middle panels show the electric‐field magnitude $|E|/E_{\max}$ (shared color scale), whereas the right panel displays the absolute error $\left|E_{\text{pred}}-E_{\text{gt}}\right|$ (separate scale). The predicted field closely reproduces the interference pattern and lobe structure observed in the reference solution, with small residual errors largely confined to regions of rapid spatial variation near the patterned surface and along fine fringes.

**FIGURE 6:**
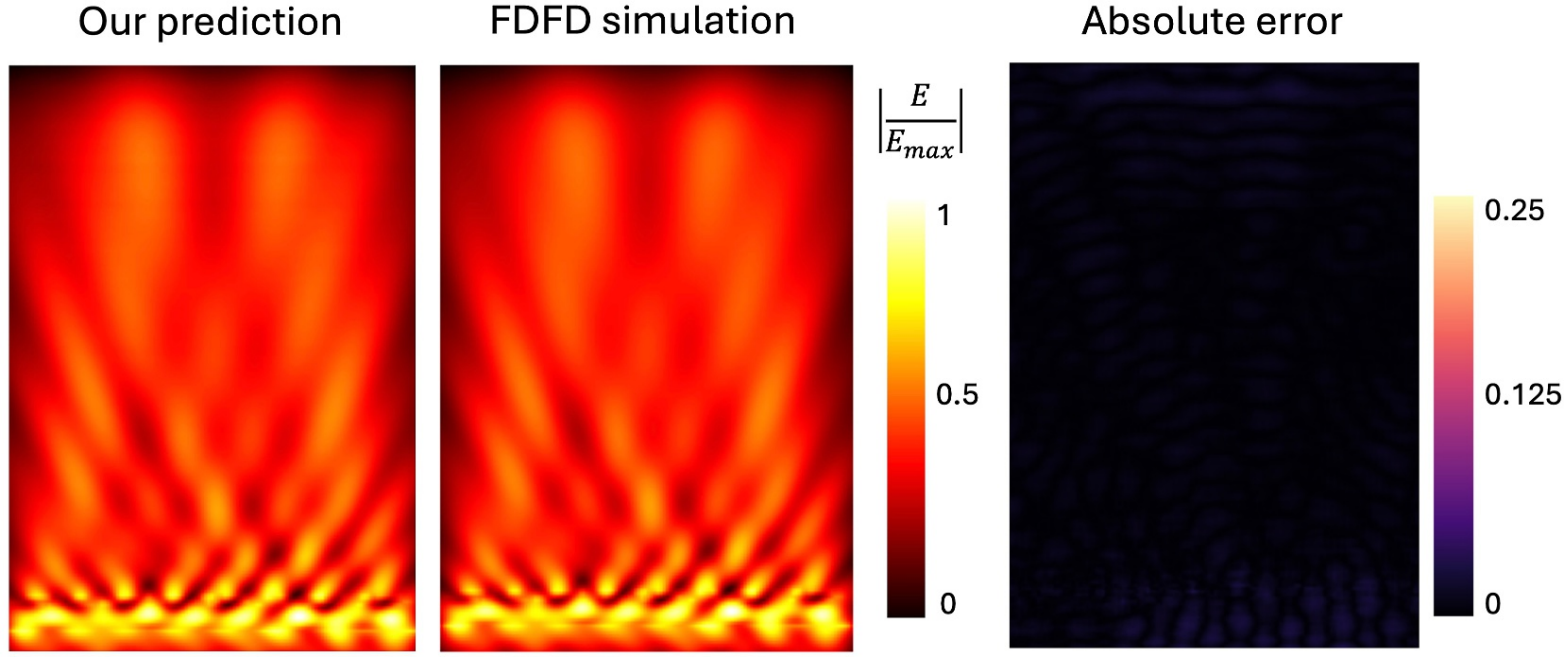
Single‐layer metasurface. Side‐by‐side comparison of the full‐wave (ground‐truth) electric‐field distribution and the field predicted by the proposed model for the single‐layer metasurface configuration.

Figure 7 visualizes absolute error maps $\left|E_{\text{pred}}-E_{\text{gt}}\right|$ at the unseen wavelength λ=410nm for the single‐layer metasurface. The five panels compare prior baselines (Refs. [17, 32, 33, 55, 56]) with the proposed model under a shared color scale (right; larger values indicate higher error). The orange dashed line marks the boundary of the design region (labeled “Design space”). Relative to the baselines, the proposed model exhibits uniformly lower errors across the domain with residuals largely confined near the device and fine interference fringes, indicating stronger generalization at wavelengths not used for training.

**FIGURE 7:**
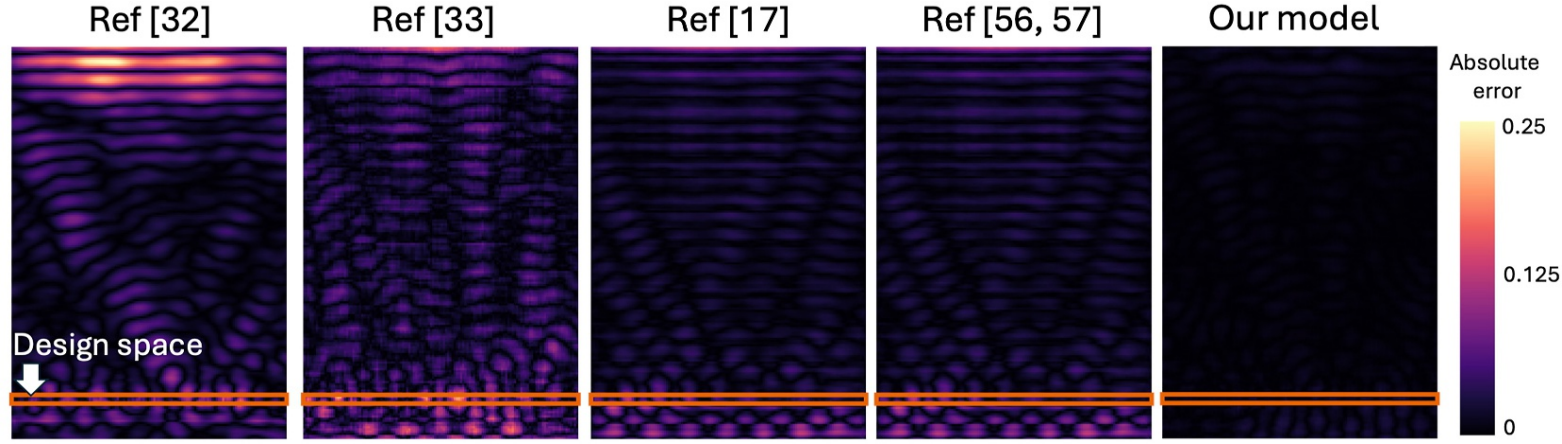
Absolute error at an unseen wavelength. Absolute error maps for the single‐layer metasurface at λ=410nm, an unseen wavelength that exhibits the largest prediction error across the evaluated band (computed as $\left|E_{\text{pred}}-E_{\text{gt}}\right|$).

Figure 8 plots the normalized mean squared error (NMSE) versus wavelength for the single‐layer metasurface, comparing the proposed method with prior baselines (refs. [17, 32, 33, 55, 56]) over the full simulation domain. Green dashed vertical lines mark the wavelengths included during training; all other wavelengths are evaluated without retraining. Curves show the mean NMSE and shaded regions indicate ±2 standard deviations. Across the 400–700 nm band, the proposed model maintains consistently low error on both trained and untrained wavelengths, whereas baselines exhibit larger fluctuations and pronounced error spikes at certain wavelengths, evidencing stronger broadband generalization of our approach.

**FIGURE 8:**
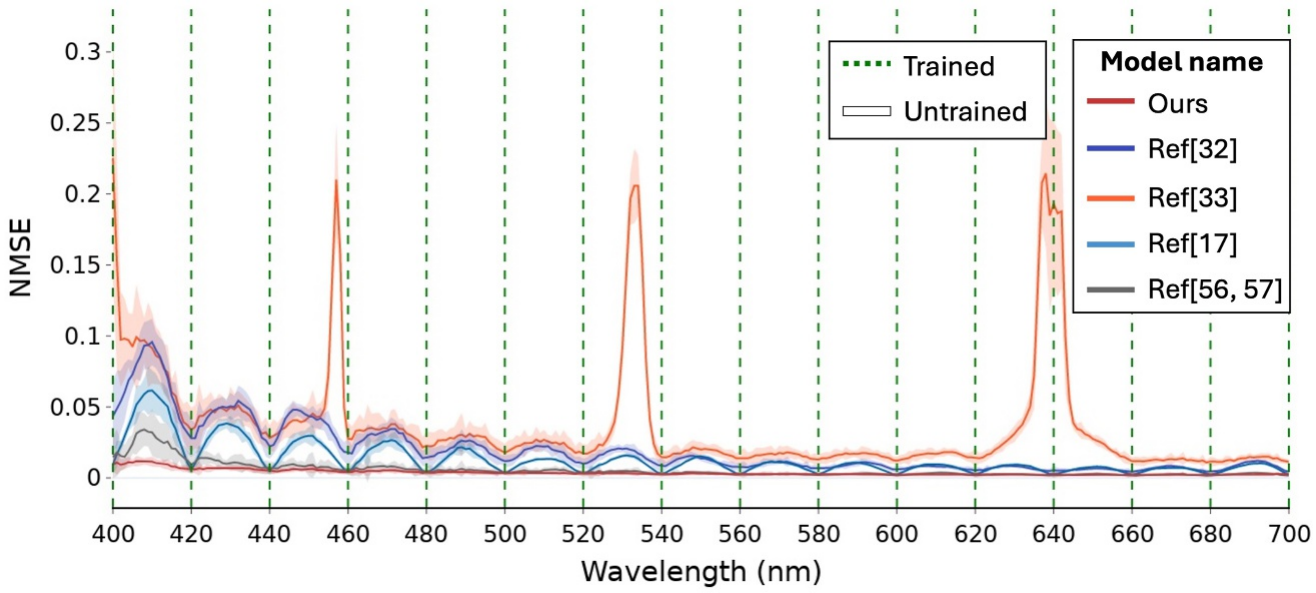
Broadband evaluation on the single‐layer metasurface. Comparison of all methods across the continuous broadband spectrum within the targeted bandwidth, evaluated over the full simulation domain. Shaded regions denote ±2 standard deviations for visual clarity, and green dashed lines mark the wavelengths used during training.

Table 1 compares single‐layer metasurface field prediction across five models. Entries show the average error (lower is better) over two spatial regions, the entire field and the structure (design) region, and for two evaluation regimes: wavelengths used during training (“Trained”) and untrained wavelengths. Our method attains the lowest error on both untrained settings (0.002035 for the entire field and 0.003812 for the structure field), reducing error by ∼39% relative to the best baseline in each column (UNet) and by ∼80% relative to NeurOLight. On trained wavelengths, our model remains competitive (0.001774 entire field; 0.003929 structure field), within 2%–4% of UNet while outperforming FNO/F–FNO and NeurOLight by 10%–13%, showing superior generalization capability of our approach.

**TABLE 1:** Comparisons of single‐layer metasurface field prediction.

Model	Entire field (absolute error)		Structure field (absolute error)	
	Trained	Untrained	Trained	Untrained
UNet	0.001732	0.003332	0.003775	0.006190
FNO	0.007719	0.013300	0.025067	0.028339
F–FNO	0.022040	0.033090	0.037091	0.043035
NeurOLight	0.001973	0.010730	0.004526	0.019503
Ours	0.001774	0.002035	0.003929	0.003812

### 3.2 Multi‐Layer Metasurface: Spectrum Splitter

Although metasurfaces offer arbitrary wavefront control, their meta‐atoms exhibit inherently frequency‐dependent resonances that reduce efficiency outside their design wavelength [57]. This bandwidth limitation restricts critical imaging applications, such as achromatic metalenses [50] and spectral beam splitters [58], to narrow operating ranges. One promising solution is a volumetric, multilayer metasurface architecture, where stacking successive patterned layers increases the effective design volume [59, 60], enabling tailored phase and amplitude responses over a wider spectrum and bringing performance closer to that of conventional bulky optics.

Although multilayer metasurfaces remain uncommon in the infrared and visible regimes, primarily due to the fabrication complexity of volumetric architectures, recent advances in two‐photon polymerization lithography [61, 62] have enabled their realization. To demonstrate the versatility of our approach, we apply our broadband surrogate model to a representative multilayer metasurface, thereby validating its applicability to complex optical structures.

#### 3.2.1 Problem Setup

Figure 9 summarizes the multilayer metasurface problem and simulation configuration. The computational domain is 5.2μm wide, 3.5μm tall, whereas the design region (blue) is embedded with a ${SiO}_{2}$ substrate (green), a normally incident source placed 1.0μm below the design region (red), and perfectly matched layers (gray) on the boundaries. This multilayer metasurface simulation environment is directly applicable to real‐world applications, such as spectrum splitters [58, 63], diffraction gratings [64, 65], LiDAR sensors [43], and many others. The design region includes five layers, each 0.7μm thick, patterned across a 3.2μm lateral span using alternating $\varepsilon_{\text{low}}$, $\varepsilon_{\text{high}}$ materials; the minimum design unit (pixel) has a width of 25nm. This setup specifies the geometry, materials, and boundary conditions used in the multilayer metasurface experiments.

**FIGURE 9:**
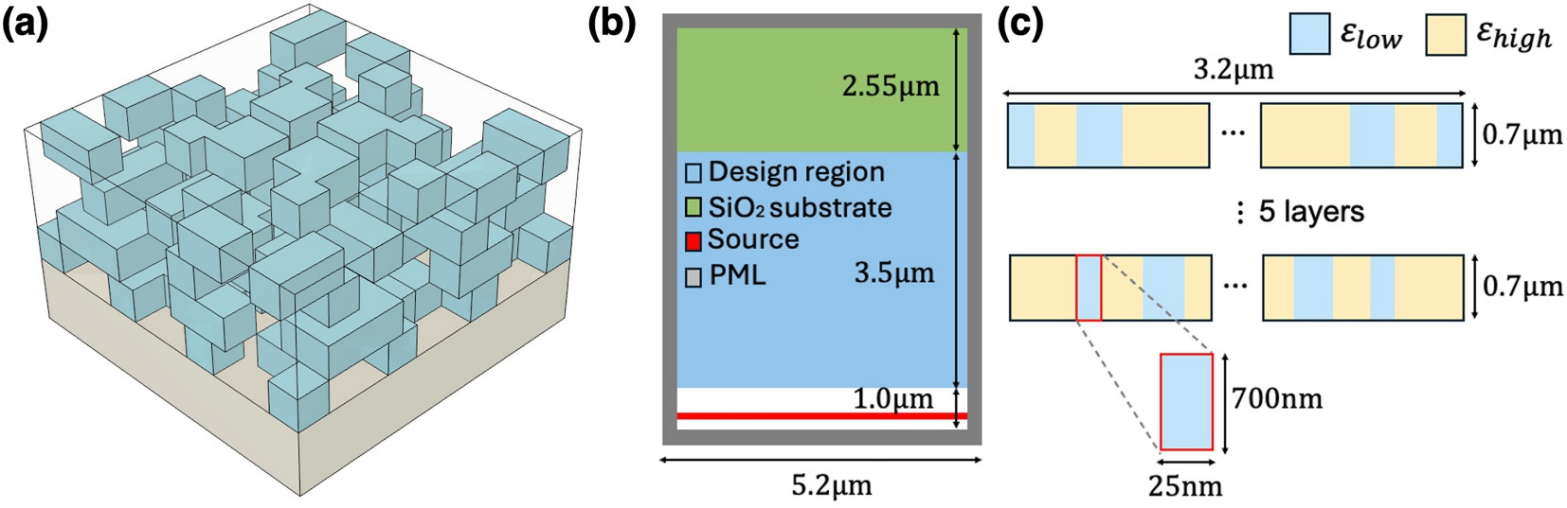
Multilayer metasurface: geometry and simulation setup. (a) Schematic of the multilayer metasurface. (b) Simulation configuration for the multilayer device. (c) Detailed view of the design region and its parameterization.

#### 3.2.2 Result

Figure 10 compares the proposed model's prediction against the full‐wave FDFD result for the multilayer metasurface and visualizes the resulting absolute error. The left and middle panels show the normalized electric‐field magnitude $|E|/E_{\max}$ under a shared color scale, whereas the right panel reports the absolute error $\left|E_{\text{pred}}-E_{\text{gt}}\right|$ with an independent scale. The prediction closely reproduces the interference fringes and scattering features present in the reference solution. The overall low error indicates high‐fidelity agreement between the surrogate and the full‐wave solver.

**FIGURE 10:**
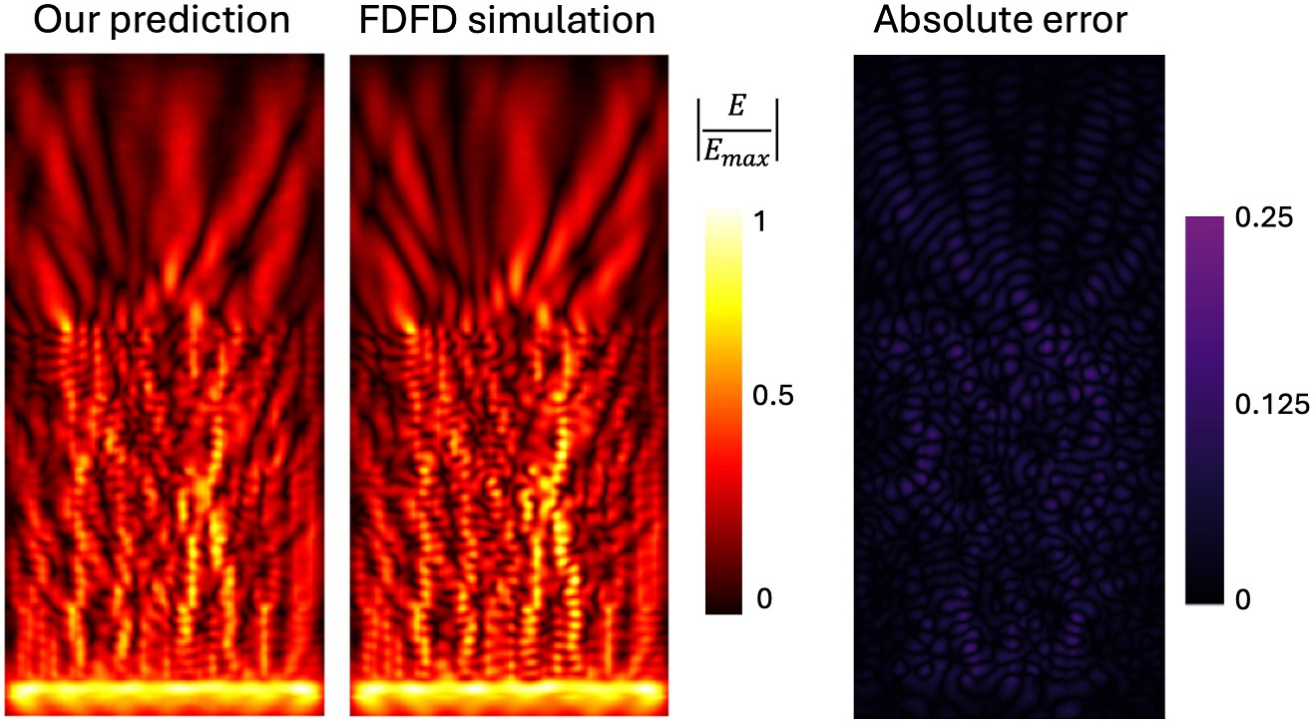
Multilayer metasurface. Side‐by‐side comparison of the full‐wave (ground‐truth) electric‐field distribution and the field predicted by the proposed model in the multilayer metasurface simulation.

Figure 11 visualizes absolute error maps $\left|E_{\text{pred}}-E_{\text{gt}}\right|$ for the multilayer metasurface at the untrained wavelength λ=410nm, which yields the largest error across the evaluated band. The panels compare prior baselines (refs. [17, 32, 33, 55, 56]) with the proposed model using a shared color scale (right); the orange box indicates the design region. Relative to the baselines, our method shows consistently lower errors both inside the device and in the surrounding field with residuals mainly confined to fine interference fringes, evidence of stronger generalization to unseen wavelengths.

**FIGURE 11:**
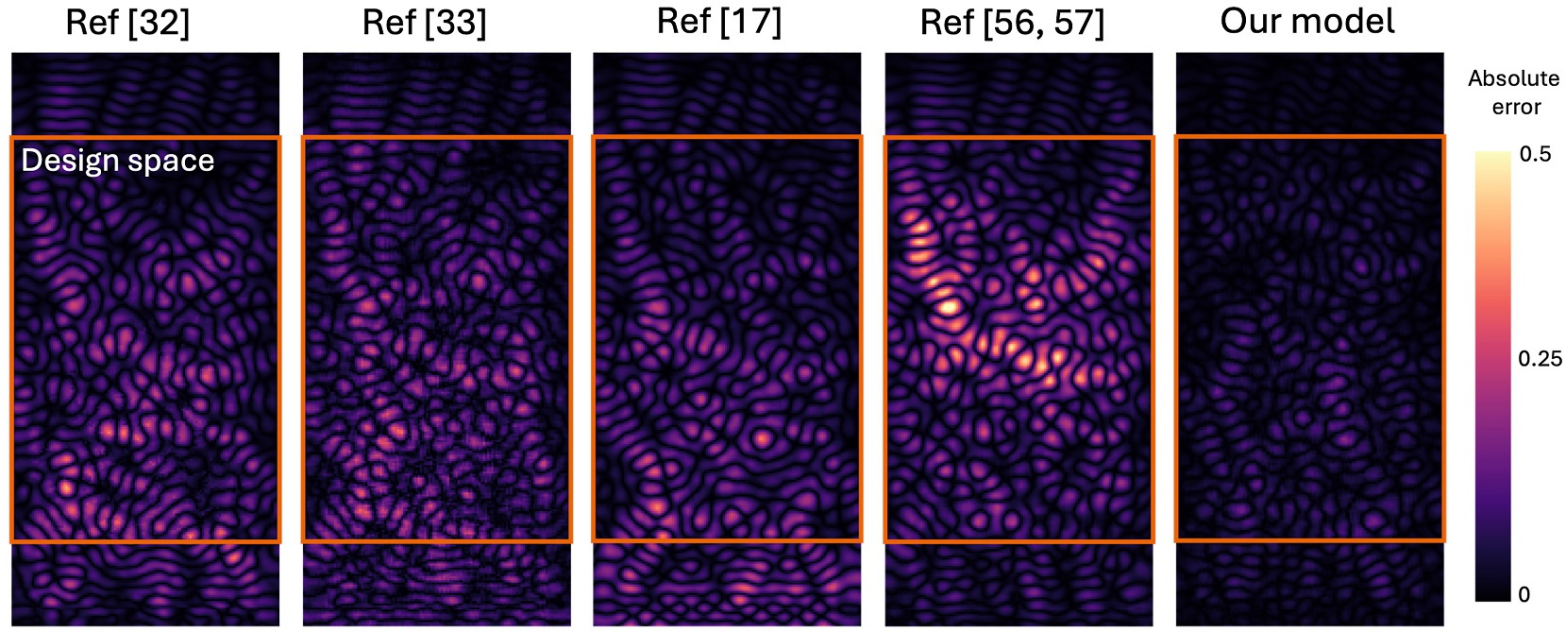
Absolute error at an unseen wavelength. Absolute error maps for the multilayer metasurface at λ=410nm—a wavelength not used in training that exhibits the largest prediction error over the evaluated band (computed as $\left|E_{\text{pred}}-E_{\text{gt}}\right|$).

Figure 12 plots NMSE versus wavelength for the multilayer metasurface, comparing the proposed method with prior baselines over the full simulation domain. Green dashed vertical lines mark wavelengths included during training; all other wavelengths are evaluated without retraining. Solid curves show mean NMSE across test structures, and shaded bands indicate ±2 standard deviations. Across the 400–700 nm range, our model sustains the lowest errors on both trained and unseen wavelengths and exhibits reduced variance, whereas baselines display larger fluctuations and pronounced error peaks, indicating a stronger broadband generalization of the proposed approach.

**FIGURE 12:**
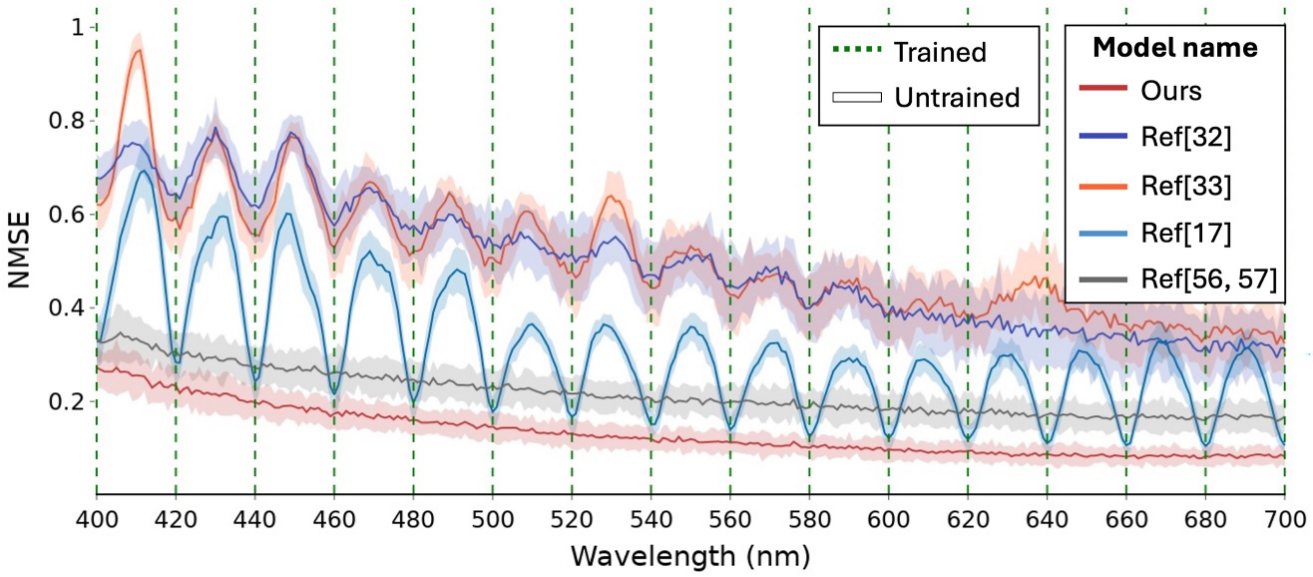
Broadband evaluation on the multilayer metasurface. Comparison of all methods across the continuous broadband spectrum within the targeted bandwidth, evaluated over the full simulation domain. Shaded regions denote ±2 standard deviations for visual clarity, and green dashed lines mark the wavelengths used during training.

Table 2 shows NMSE (lower is better) for multilayer metasurface field prediction, averaged over the evaluated band. Results are shown for two spatial regions, the entire field and the structure (design) region, and for two evaluation regimes: wavelengths included during training (“Trained”) and untrained wavelengths. Our method achieves the lowest error in all four settings (Entire: 0.1360 trained/0.1329 untrained; Structure: 0.1549 trained/0.1515 untrained), outperforming UNet, FNO, F–FNO, and NeurOLight. Notably, although some baselines degrade on unseen wavelengths (e.g., NeurOLight), our model maintains nearly unchanged error between trained and untrained evaluations, indicating stronger broadband generalization. In conclusion, this demonstrates that our method can generalize reliably across wavelengths, even in environments where the spectral consistency assumption is significantly weakened.

**TABLE 2:** Comparisons of multilayer metasurface field prediction.

	Entire field (absolute error)		Structure field (absolute error)	
	Trained	Untrained	Trained	Untrained
Unet	0.2182	0.2167	0.2013	0.1987
FNO	0.4712	0.4962	0.5055	0.5184
F‐FNO	0.4579	0.4579	0.4730	0.5143
NeurOLight	0.1683	0.3160	0.1949	0.2785
Ours	0.1360	0.1329	0.1549	0.1515

### 3.3 Freeform Waveguide: Photonic‐Integrated Circuit

Integrated photonics [66, 67] has emerged as a leading platform for next‐generation high‐performance computing, leveraging light's intrinsic advantages, low latency, immense bandwidth, and multiplexing capabilities. Advances in photonic integrated circuits (PICs), which integrate a diverse array of optoelectronic components on a single chip, have paved the way for photonic–electronic co‐integrated systems. These hybrid circuits hold great promise as hardware accelerators for both conventional high‐performance computing and emerging AI workloads. Recent advances in photonic integrated circuits have demonstrated on‐chip optical tensor convolution [14, 68], enabling fully optical neural networks that can dramatically accelerate convolutional operations.

Adjoint‐based inverse design has become one of the prevailing standards in PIC optimization because it efficiently computes gradients of performance metrics. However, each optimization step still requires full‐wave Maxwell solutions, so a single device often demands hundreds of electromagnetic simulations. As a result, adjoint methods have been largely confined to individual components of PICs (e.g., wavelength demultiplexers [66], mode converters [69, 70], or grating couplers [71, 72]). Here, we leverage our broadband surrogate model to predict the field profile of the freeform waveguide, achieving grid‐size precision while significantly reducing the computational cost.

#### 3.3.1 Problem Setup

Figure 13 summarizes the simulation setup of the freeform waveguide problem. It has a 5.2μm×8.85μm simulation domain. Polymer regions ($\varepsilon_{r}$ = 2.25) and air are arranged in 25 nm‐scale pixels within an unconstrained design region.

**FIGURE 13:**
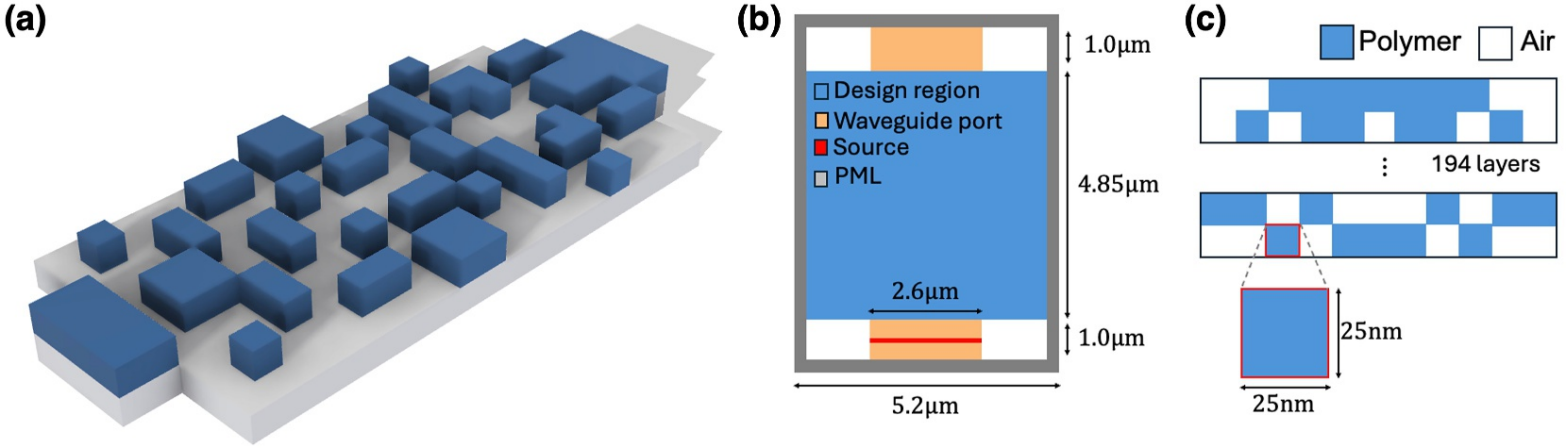
Freeform waveguide: geometry and simulation setup. (a) Schematic of the freeform waveguide. (b) Simulation configuration for the freeform device. (c) Detailed view of the design region and its parameterization.

#### 3.3.2 Result

Figure 14 compares the surrogate's prediction with the full‐wave FDFD result for the freeform waveguide and visualizes the residual error. Although this simulation setting includes a relatively smaller permittivity contrast compared to the spectrum splitter simulation, the unconstrained geometric design causes complex electromagnetic phenomena. The left and middle panels display the normalized electric‐field magnitude $|E|/E_{\max}$ under a shared color scale, whereas the right panel shows the absolute error $\left|E_{\text{pred}}-E_{\text{gt}}\right|$ with its own scale. The prediction closely reproduces the interference fringes and scattering features present in the reference. The uniformly low error indicates high‐fidelity agreement between the surrogate and the full‐wave solver.

**FIGURE 14:**
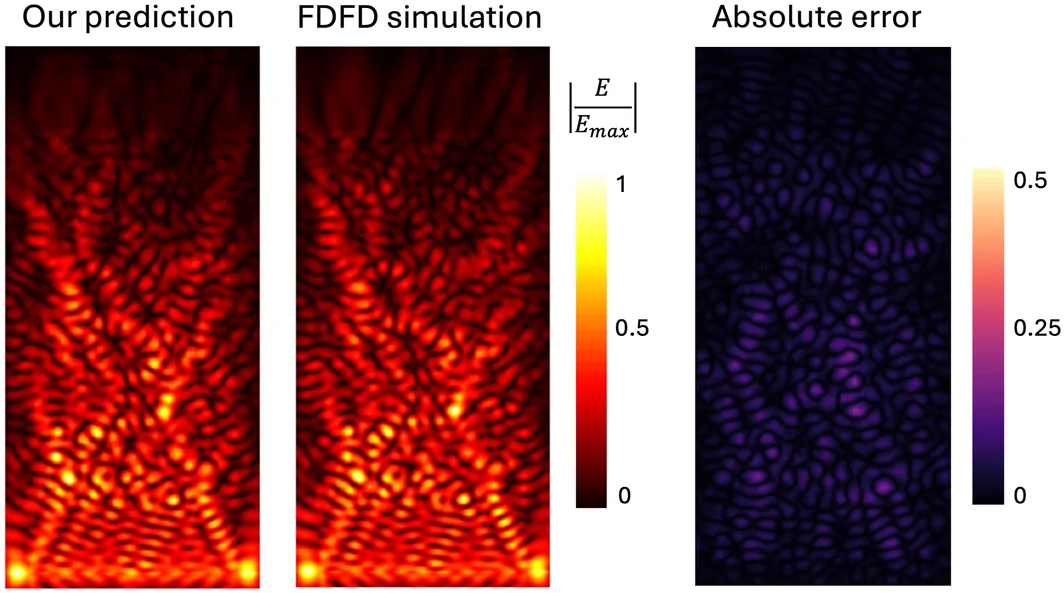
Freeform waveguide. Comparison of the full‐wave (ground‐truth) electric‐field distribution and the field predicted by the proposed model for the freeform waveguide configuration.

Figure 15 visualizes absolute error maps $\left|E_{\text{pred}}-E_{\text{gt}}\right|$ for the freeform waveguide at the untrained wavelength λ=410nm, which yields the largest error across the evaluated band. The panels compare prior baselines (refs. [17, 32, 33, 55, 56]) with the proposed model under a shared color scale (right); the orange rectangle delineates the design region (“Design space”). Relative to the baselines, our method exhibits visibly lower errors both inside the device and in the surrounding field.

**FIGURE 15:**
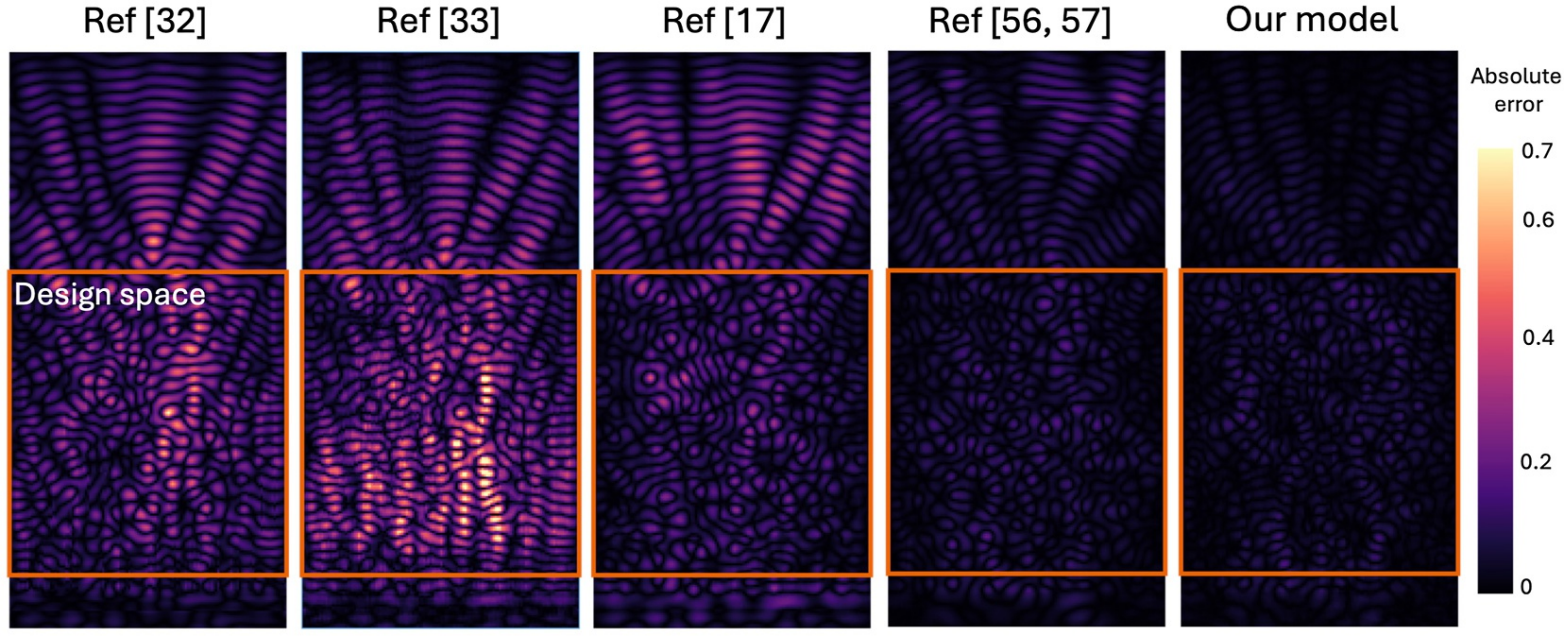
Absolute error at an unseen wavelength. Absolute error maps for the freeform waveguide at λ=410nm—a wavelength not used in training that exhibits the largest prediction error across the evaluated band (computed as $\left|E_{\text{pred}}-E_{\text{gt}}\right|$).

Figure 16 plots NMSE versus wavelength for the freeform waveguide, comparing the proposed method with prior baselines over the full simulation domain. Green dashed vertical lines mark wavelengths included during training; all other wavelengths are evaluated without retraining. Solid curves report the mean NMSE across test structures, and shaded bands indicate ±2 standard deviations. Across the 400–700 nm range, our model maintains the lowest errors on both trained and unseen wavelengths and exhibits reduced variance, whereas baselines show larger fluctuations and pronounced error peaks, evidencing stronger broadband generalization.

**FIGURE 16:**
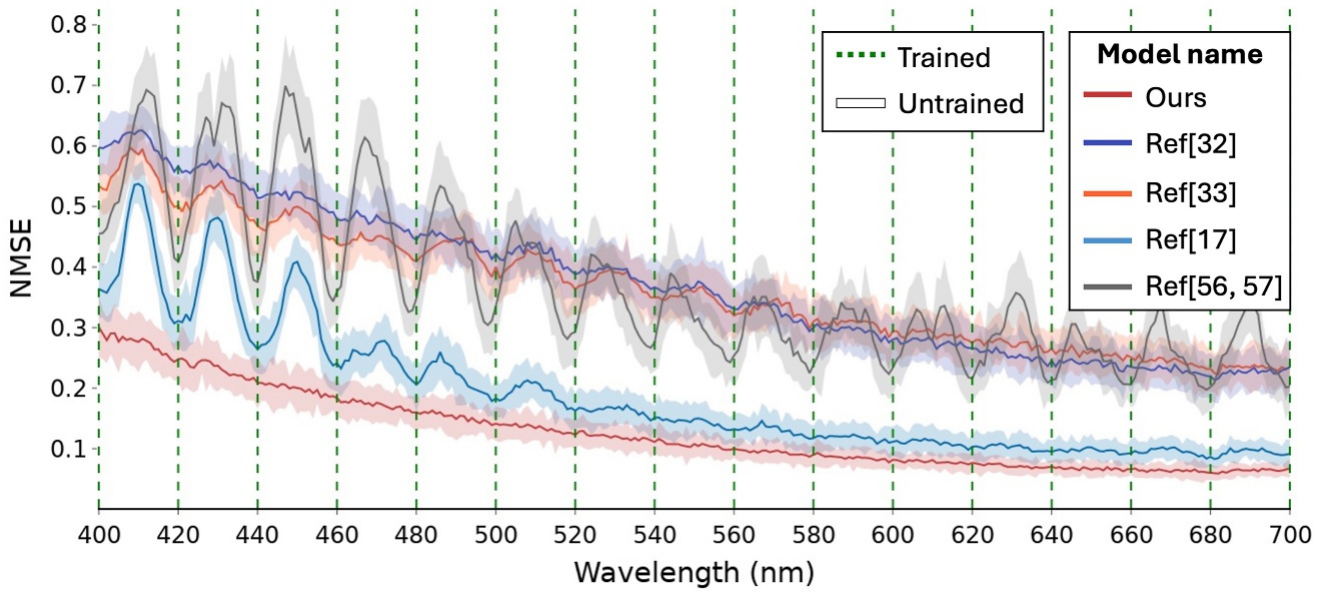
Broadband evaluation on the freeform waveguide. Comparison of all methods across the continuous broadband spectrum within the targeted bandwidth, evaluated over the full simulation domain. Shaded regions denote ±2 standard deviations for visual clarity, and green dashed lines mark the wavelengths used during training.

Table 3 shows NMSE for freeform waveguide field prediction across two spatial regions, the entire field and the design region, and two evaluation settings: wavelengths seen during training (“Trained”) and untrained wavelengths. Our method attains the lowest error in all four cases (Entire: 0.1305 trained/0.1273 untrained; design: 0.1528 trained/0.1479 untrained), improving over the strongest baseline, NeurOLight, by ≈23%–33% depending on the setting. The minimal trained–untrained gap—indeed a slight decrease on unseen wavelengths—indicates robust broadband generalization, whereas UNet, FNO, and F–FNO exhibit substantially higher errors, particularly within the design region.

**TABLE 3:** Comparisons of freeform waveguide field prediction.

	Entire field (absolute error)		Design field (absolute error)	
	Trained	Untrained	Trained	Untrained
Unet	0.2886	0.3723	0.3294	0.4211
FNO	0.3681	0.3735	0.4296	0.4313
F‐FNO	0.3529	0.3650	0.4140	0.4193
NeurOLight	0.1696	0.1892	0.1996	0.2124
Ours	0.1305	0.1273	0.1528	0.1479

Despite the increased complexity of the freeform structure, the proposed model still achieves the lowest average NMSE and error variance across the simulation region. In particular, the error increase for untrained wavelengths remains minimal, further validating the model's generalization ability. In contrast, the other models exhibit both higher average errors and broader error distributions. The results also demonstrate that our method enables robust electromagnetic field prediction even under the weakened spectral consistency assumption by a highly complex electromagnetic interplay from structurally unconstrained design conditions.

### 3.4 Computational Efficiency

#### 3.4.1 Evaluating Performance Across Varying Dataset Sizes

To analyze the dependency between dataset size and model performance, we conduct additional experiments evaluating prediction accuracy across various training dataset sizes. As shown in Figure 17, our method consistently outperforms the baseline models for untrained wavelengths across all dataset sizes, including significantly reduced size. These results demonstrate the data efficiency of our approach. Such data‐efficient performance has practical implications for applications where data collection is expensive or limited.

**FIGURE 17:**
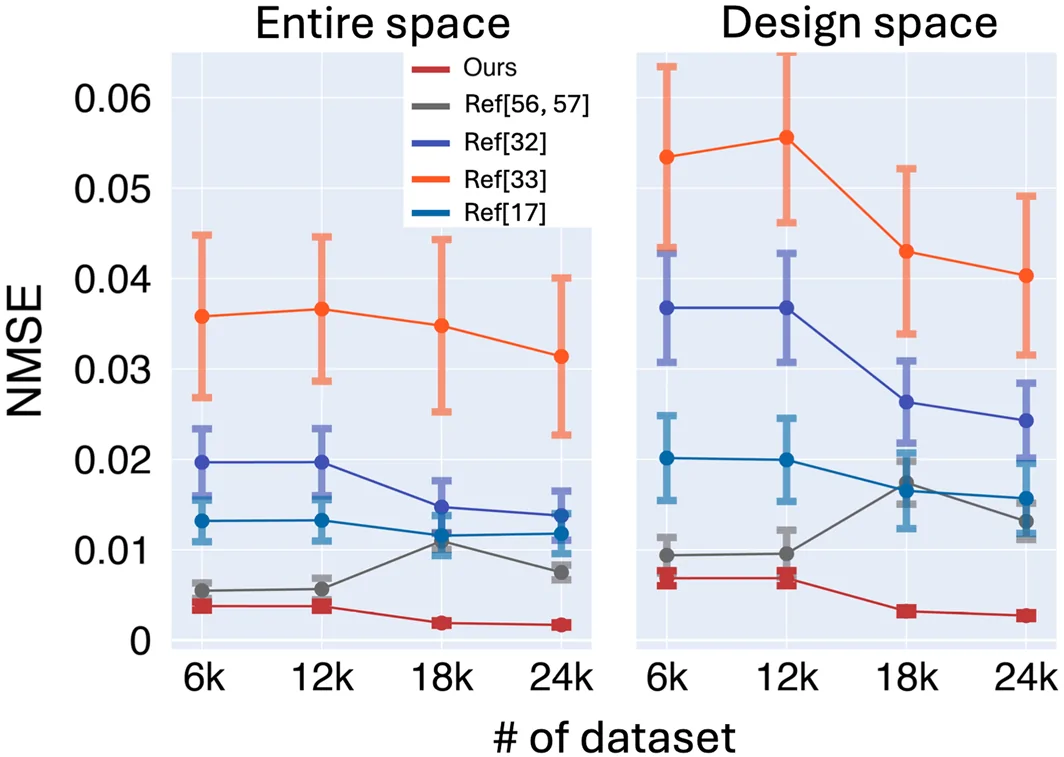
Dataset size ablation on the single‐layer metalens. Field‐prediction accuracy as a function of training‐set size, reported for wavelengths included during training (Trained) and for untrained wavelengths (Unseen). Lower values indicate better performance. Whiskers denote the standard deviation scaled by 1/5 for visual clarity.

#### 3.4.2 Computational Efficiency and Model Compactness

Our method also delivers substantial acceleration relative to a conventional numerical solver. To assess computational efficiency and model compactness, we compare per‐sample runtime between the FDFD simulator (Ceviche [54]) and our surrogate solver (Table 4). With a batch size of 1, our model achieves ∼16× speedup; under the default batch size of 32, the speedup increases to ∼42×, representing an order‐of‐magnitude improvement in runtime efficiency over the numerical solver.

**TABLE 4:** Runtime per sample.

	Ours (batch size 1)	Ours (batch size 32)	Numerical simulation
Time (s)	0.0172	0.0067	0.2786

*Note:* Computational time per sample for the proposed surrogate compared with a conventional finite‐difference frequency‐domain (FDFD) solver (Ceviche). Field prediction with our model was performed on a single RTX 6000 Ada GPU, whereas the FDFD simulation ran on a 64‐core AMD 5995WX CPU.

Beyond runtime, we analyze parameter complexity (Table 5). In 2D, a standard FNO layer requires $C^{2}M_{v}M_{h}$ parameters, where $M_{v}$ and $M_{h}$ are the numbers of retained frequency modes in the vertical and horizontal directions, respectively, and C is the channel count, leading to large models. In contrast, our surrogate uses only 0.43 million parameters, ≈86.9% reduction relative to FNO‐2D [32]'s 3.29 million and ≈96.33% reduction compared with UNet [55, 56]'s 11.60 million. This reduction stems from our *Fourier Group Convolutional Shuffling*, which decomposes each Fourier layer into separate horizontal and vertical transforms and applies group‐wise weight sharing with channel shuffling, compressing the spectral weight tensor from $C^{2}M_{v}M_{h}$ to $C^{2}\left(M_{v}+M_{h}\right)/\left(4G\right)$, where G is the number of groups. Despite the substantially smaller parameter count, the model attains state‐of‐the‐art accuracy in both the design and propagation regions.

**TABLE 5:** Comparison of the number of parameters across models.

Model	Parameters (M)
U‐Net	11.60
FNO	3.29
F‐FNO	1.89
NeurOLight	1.65
Ours	0.43

### 3.5 Limitations and Future Directions

Although the proposed physics‐guided neural operator demonstrates robust performance and a highly compact architecture across diverse nanophotonic platforms, certain limitations present clear opportunities for future research. A primary simplification in our current baseline test cases is the assumption of constant, nondispersive refractive indices. In physical broadband applications, material dispersion and loss are dominant effects that dictate the lineshape and spectral position of optical resonances. Although our extended analyses indicate that the model's predictive capability remains stable across a wide range of arbitrary permittivities, incorporating wavelength‐dependent complex refractive indices into the conditional embedding is an essential next step.

Furthermore, it is important to clarify that the current study demonstrates high‐fidelity continuous‐spectrum interpolation within a targeted operational bandwidth (400–700 nm), rather than unbounded spectral extrapolation outside the training range. Although interpolating across this wide fractional bandwidth is a substantial achievement, predicting electromagnetic phenomena entirely outside the trained domain remains an active challenge for data‐driven solvers. Nevertheless, by leveraging the intrinsic scale invariance of Maxwell's equations, the interpolative capabilities demonstrated here can be mathematically mapped to different physical bandwidths using scaling factors, thereby broadening the model's applicability without requiring direct spectral extrapolation.

Another important consideration is the training data sampling strategy. The current model relies predominantly on fixed uniform spectral sampling (20 nm gaps) to systematically validate the architectural contributions of the proposed physics‐guided priors. However, the ability to perfectly resolve highly abrupt spectral features, such as sharp resonances, is intrinsically tied to this sampling distribution. Future studies investigate optimal data acquisition strategies, including sparse, nonuniform, random, or Chebyshev sampling, to determine the most data‐efficient methods for capturing complex spectral landscapes while avoiding the harmonic blind spots inherent to uniform grids [73].

Finally, although the proposed model exhibits orders‐of‐magnitude acceleration compared to single‐instance FDFD calculations, a more comprehensive computational benchmark is necessary for a holistic assessment. In practical broadband nanophotonics design, conventional workflows often utilize single‐shot FDTD simulations or highly parallelized FDFD runs distributed across multi‐node clusters. Future work will benchmark the proposed neural operator against these massively parallel and inherently broadband numerical environments to definitively quantify its computational advantages in true industrial and academic workflows.

## 4 Conclusion

In this paper, we present an electromagnetic surrogate solver that generates highly accurate, full‐wave solutions to Maxwell's equations over a continuous, broadband spectrum, even when the training set contains only sparsely sampled wavelengths. Our model inherently maintains strong predictive accuracy at intermediate wavelengths not seen during training, thereby overcoming the generalization limitations of existing surrogate solvers in electromagnetism. Moreover, by exploiting the intrinsic scale invariance of Maxwell's equations [38], the solutions obtained at one frequency can be uniformly extended to any other target frequency band through simple multiplication by an appropriate scaling factor.

To ensure broad‐spectrum generalizability, we introduce two foundational concepts. First, spectral consistency, which formalizes the inherent link between wavelength‐dependent field variations and spatial frequency as dictated by Maxwell's equations. Second, a conditional embedding framework supported by an efficient conditioning mechanism. Concretely, we use the polarization‐dependent wave prior and a wave‐informed element‐wise multiplicative encoding, each explicitly crafted to enforce spectral consistency. By uniting these components, our solver produces reliable, high‐fidelity predictions at wavelengths beyond those seen during training, thereby overcoming the shortcomings of prior surrogate approaches and markedly advancing the generalization capabilities of deep‐learning‐based electromagnetic simulators. We evaluate our surrogate solver on three representative structures, namely a single layer metasurface, a multilayer metasurface, and a freeform waveguide, each with distinct operating mechanisms and geometric complexity. Across their target bandwidths, our model reduces average electric‐field prediction error relative to the state of the art [17] by 80.3%, 56.8%, and 32.3% for three examples, and lowers model complexity by 86.9% compared with FNO2d [32]. Extension of our framework to macroscopic length scales is attainable through integration with emerging fast‐solver techniques [74, 75, 76]. Although enlarging the simulation domain naturally raises concerns about model complexity and training efficiency, contemporary deep‐learning architectures comprising hundreds of millions—or even billions—of parameters can be trained effectively on modern hardware. Consequently, developing a large‐scale, three‐dimensional extension of our surrogate solver represents a significant advance toward replacing conventional numerical methods for solving Maxwell's equations.

## Author Contributions

**Joonhyuk Seo:** conceptualization, methodology, software, data curation, validation, visualization, writing – review and editing, writing – original draft, investigation, formal analysis. **Chanik Kang:** conceptualization, methodology, software, data curation, validation, visualization, writing – original draft, writing – review and editing, investigation, formal analysis. **Dongjin Seo:** conceptualization. **Haejun Chung:** supervision, funding acquisition, visualization, writing – review and editing, project administration, validation, writing – original draft.

## Conflicts of Interest

The authors declare no conflicts of interest.

## Supporting information


Supporting Information S1

## Data Availability

All data supporting the findings of this study are available within the article and its Supporting Information S1. Source data are provided with this paper. And the photonic structure datasets are available at https://drive.google.com/drive/folders/1YRuzbOaIv3q6AjBgvagha2H2cizaP1Rh?usp=sharing. The code used for the experiments presented in this paper is available at https://github.com/yhy258/WINO_interp.
