# Supplementary material for: Broadband Electromagnetic Field Prediction at Unseen Wavelengths via Physics‐Guided Neural Operators
Source: Nanophotonics. 2026 Aug 9;15(15):e70242. doi: 10.1002/nap2.70242 (PMC13642526; doi:10.1002/nap2.70242)
Supplement: Supplementary file 1 [file NAP2-15-e70242-s001.pdf]

Supplementary Material

Broadband Electromagnetic Field Prediction at Unseen Wavelengths via Physics-Guided Neural Operators

Joonhyuk Seo[1†], Chanik Kang[2†], Dongjin Seo[3], Haejun Chung[2*]

[1]Department of Electrical Engineering and Computer Science, University of California Irvine, Irvine, USA.

[2*]Department of Artificial Intelligence, Hanyang University, 222 Wangsimni-ro, 04763, Seoul, Republic of Korea.

[3]Department of Applied Physics, Yale University, 55 College St, Connecticut, 06520, New Haven, USA.

*Corresponding author(s). E-mail(s): haejun@hanyang.ac.kr;
Contributing authors: joonhys1@uci.edu; chanik@hanyang.ac.kr; ;
†These authors contributed equally to this work.

1 Related Works

This section reviews recent advancements in physics-informed surrogate solvers in the electromagnetics area, with a primary focus on meural metwork-based approximations and operator learning.

Neural Network-Based Approximations. Prior work on electromagnetic field prediction has largely trained neural networks to regress simulated electric and magnetic fields. Data-driven approaches [1–3] sought to learn surrogates of Maxwell's equations from simulated field data, but they often failed to encode key physical structure. To mitigate this gap, PINNs were introduced within a physics-augmented deep learning framework [4], combining a data-fidelity loss with a Maxwell-residual loss to embed the governing equations during training. Although this strategy accelerates inference relative to conventional FDFD solvers, it remains constrained: a distinct dataset is required for each wavelength, predictions are limited to the wavelengths

seen during training, and reported demonstrations are confined to relatively small, geometrically simple photonic structures.

Advancements in Operator Learning. Purely data-driven models have struggled to faithfully capture the structure of Maxwell's equations, motivating a shift toward neural operator frameworks that model mappings between function spaces for complex physical systems [5, 6]. Recent advances in operator learning provide discretization-invariant representations that more effectively encode PDE-governed dynamics. FNOs [6] learn mappings between infinite-dimensional function spaces via spectral convolutions, yielding efficient and accurate PDE solvers. FNOs exhibit resolution invariance and support zero-shot super-resolution, establishing them as a foundational approach in operator learning. Building on this, factorized FNO (F-FNO) [7] employs separable spectral layers and strengthened residual connections, substantially reducing error across diverse PDE benchmarks. In electromagnetic field prediction, the state-of-the-art model, NeurOLight [8], adopts an FNO backbone and encodes a wave prior by combining wavelength information with material permittivity, achieving superior parameter efficiency and accuracy over earlier surrogates. Nevertheless, NeurOLight remains constrained to a narrow range of wavelengths and spatial discretizations (grid steps), limiting its suitability for broadband applications.

2 Background and Motivation

2.1 Fundamentals of the Electromagnetic Simulations

Light can be treated as an electromagnetic wave, and its propagation is governed by Maxwell's equations, a set of coupled PDEs describing how electric and magnetic fields evolve in space and time. Under time-harmonic conditions (with $e^{-j\omega t}$ dependence), Maxwell's equations simplify to the frequency-domain form:

$$\nabla \times \mathbf{E}(\mathbf{r}) = -j\omega\mu_0\mathbf{H}(\mathbf{r}), \tag{1}$$

$$\nabla \times \mathbf{H}(\mathbf{r}) = j\omega\epsilon_0\epsilon_r(\mathbf{r})\mathbf{E}(\mathbf{r}), \tag{2}$$

where $\mathbf{E}(\mathbf{r})$ and $\mathbf{H}(\mathbf{r})$ are the electric and magnetic fields, respectively. Here, ω is the angular frequency, μ_0 and ϵ_0 are the permeability and permittivity of free space, and $\epsilon_r(\mathbf{r})$ is the relative permittivity distribution in the medium.

By substituting (1) into (2), we eliminate one of the fields and arrive at a vector Helmholtz-type PDE in terms of $\mathbf{E}(\mathbf{r})$:

$$\nabla \times \nabla \times \mathbf{E}(\mathbf{r}) - k_0^2\epsilon_r(\mathbf{r})\mathbf{E}(\mathbf{r}) = 0, \tag{3}$$

where $k_0 = \omega\sqrt{\mu_0\epsilon_0}$ is the free-space wavenumber. This Helmholtz equation, governing the spatial distribution of the electric field, represents the PDE we seek to approximate using a surrogate solver.

To generate the training and validation datasets necessary for building our surrogate solver, we use the Finite-Difference Frequency-Domain (FDFD) method [9, 10]. FDFD discretizes the spatial domain into a grid and applies finite differences to

approximate spatial derivatives, enabling the efficient computation of steady-state electromagnetic fields for a given frequency.

3 Model Architecture

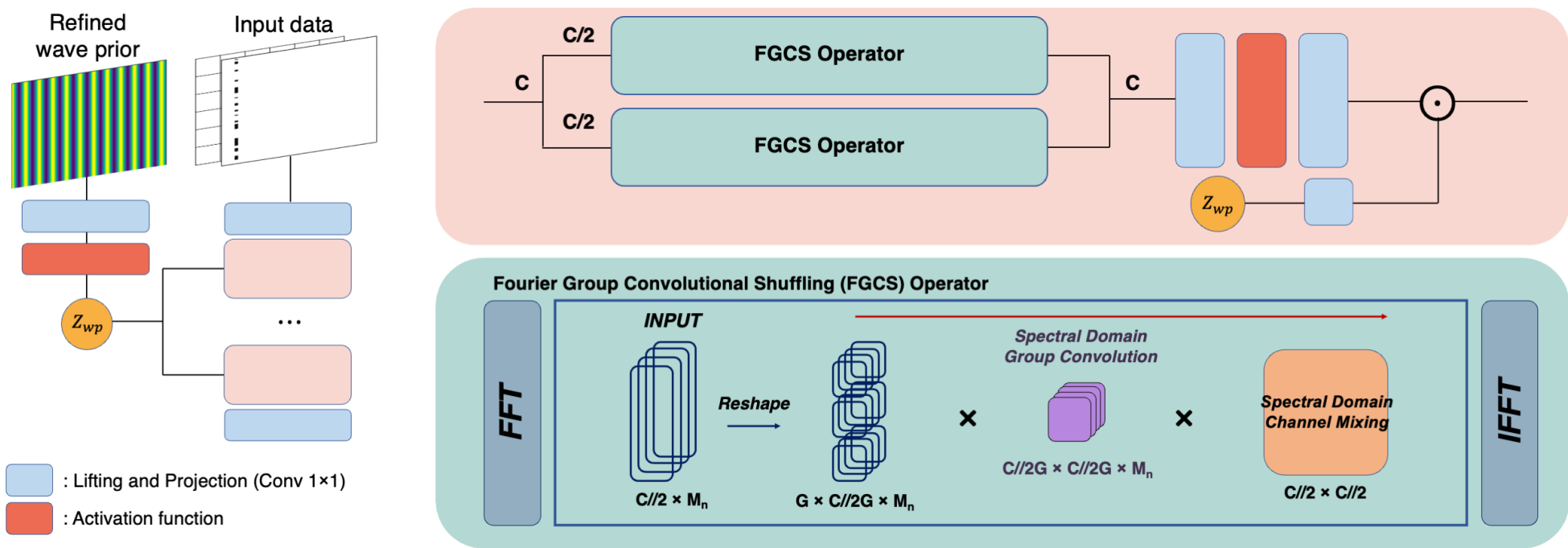

Fig. S1 Schematic illustration of (left) the proposed overall framework including (top) each layer and Wave-Informed element-wise Multiplicative Encoding (WIME) and (bottom) Fourier Group Convolution Shuffling (FGCS) operator.

Similar to a prior work [11], our model has an iterative process to map between infinite-dimensional function spaces.

$$u = \mathcal{G}_\theta(a) = (\mathcal{Q} \circ \mathcal{L}^{(L)} \circ \cdots \circ \mathcal{L}^{(1)} \circ \mathcal{P})(a) \tag{4}$$

where $\mathcal{L}^{(l)}$ is a l-th operator layer, L indicates the number of layers, and $\mathcal{P}, \mathcal{Q}$ are lifting and projection operators, respectively.
We define the layers $\mathcal{L}^{(l)}$ as follows:

$$\mathcal{L}^{(l)}(z^{(l)}) = z^{(l)} + W_2^{(l)}\sigma(W_1^{(l)}\tilde{\mathcal{K}}^{(l)}(z^{(l)}) + b_1^{(l)}) + b_2^{(l)} \tag{5}$$

where $\tilde{\mathcal{K}}^{(l)}$ is our kernel integral operator based on the Fourier transform [6] detailed in Section 2.1 in this material.

We employ a residual connection after two linear transformations with an activation function to enhance the capacity of our model, like [7, 12]. In addition, we use 1×1 convolution layers instead of 3×3 convolution layers to ensure discretization invariance, unlike a previous study [8].

3.1 Kernel Integral Operator $\tilde{\mathcal{K}}$

The kernel integral operator in the original FNO is defined as follows:

$$\mathcal{K}^{(l)} = \mathcal{F}^{-1}(R^{(l)} \cdot \mathcal{F}),\ R \in \mathbb{C}^{M_v \times M_h \times C \times C} \tag{6}$$

where $\mathcal{F}$ is a Fourier transformation, M_v, M_h, and C denote the number of frequency modes of vertical and horizontal components and the number of channels, respectively. Thus, the number of parameters in a 2D problem is $C^2 M_v M_h$. This results in a substantially high parameter complexity. Thus, as illustrated in Fig. S1 (top), we construct the cross-shaped block [8] to achieve superior parameter efficiency. We split the inputs into two chunks $z^{(l)} = [z_h^{(l)}, z_v^{(l)}]$ along the channel axis and factorize the Fourier transforms over the horizontal and vertical dimensions $\tilde{\mathcal{K}}^{(l)}(z^{(l)}) = [\mathcal{K}_h^{(l)}(z_h^{(l)}), \mathcal{K}_v^{(l)}(z_v^{(l)})]$. Therefore, the weights $R_h^{(l)} \in \mathbb{C}^{M_h \times \frac{1}{2}C \times \frac{1}{2}C}$ and $R_v^{(l)} \in \mathbb{C}^{M_v \times \frac{1}{2}C \times \frac{1}{2}C}$ of both two blocks have $\frac{C^2}{4}(M_h + M_v)$ parameters, which is significantly fewer than the $C^2 M_h M_v$ parameters in the kernel integral operator of FNO.

Furthermore, we propose an efficient Fourier Group Convolution Shuffling (FGCS) operator, which achieves high parameter efficiency while maintaining strong performance, as illustrated in Fig. S1 (top and bottom). Our approach applies grouping in the Fourier domain along the channel dimension, introducing the group parameter G in a weight-sharing manner, as inspired by [13–15].

To provide a detailed discussion of the FGCS operator, we define the Fourier transformed input feature as $v^{(l)}$ in a l-th layer. For simplicity, we only consider vertical axis FGCS operation. Thus the feature's shape would be $v^{(l)} \in \mathbb{C}^{M \times W \times \frac{1}{2}C}$.

First, we reshape the tensor $v^{(l)}$ (The symbol $\Rightarrow$ denotes a tensor reshaping notation):

$$v^{(l)} \in \mathbb{C}^{M \times W \times \frac{1}{2}C} \Rightarrow v^{(l)} \in \mathbb{C}^{M \times W \times G \times \frac{1}{2G}C} \tag{7}$$

Then, we apply matrix multiplication with a shared weight matrix $R^{(l)} \in \mathbb{C}^{M \times \frac{C}{2G} \times \frac{C}{2G}}$ in the spectral domain:

$$v'^{(l)}_{i,j,p,q} = \sum_{k=1}^{\frac{1}{2G}C} v^{(l)}_{i,j,p,k} R^{(l)}_{i,k,q} \tag{8}$$

Subsequently, we conduct an additional tensor reshaping to make the shape of feature $v'^{(l)}$ be the original tensor shape $M \times W \times \frac{1}{2}C$:

$$v'^{(l)} \in \mathbb{C}^{M \times W \times G \times \frac{1}{2G}C} \Rightarrow v'^{(l)} \in \mathbb{C}^{M \times W \times \frac{1}{2}C} \tag{9}$$

However, the weight-sharing scheme in the group convolution introduces significant information loss, leading to a substantial decline in performance. To mitigate this issue, we implement channel shuffling in the Fourier domain, which can be efficiently performed through matrix multiplication along the channel dimension using $W_{\text{ch}}^{(l)} \in \mathbb{C}^{\frac{1}{2}C \times \frac{1}{2}C}$:

$$v'^{(l)}_{i,j,p} = \sum_{k}^{\frac{C}{2}} v'^{(l)}_{i,j,k} W^{(l)}_{\text{ch}\ k,p} \tag{10}$$

The channel shuffling scheme can compensate for the lost information by weight-sharing group convolution, thereby preserving performance while achieving exceptional parameter efficiency.

3.2 Wave-Informed element-wise Multiplicative Encoding

Here, we provide detailed transformations of Wave-Informed Element-wise Multiplicative Encoding (WIME) as shown in Fig. S1.

First, we create a common embedding, which is then injected into all layers, using a 1×1 convolutional layer w^{wp} with a nonlinear activation function σ:

$$z_{i,j,p}^{wp} = \sigma(\sum_{k}^{2} \mathcal{W}_{i,j,k} w_{k,p}^{wp}) \tag{11}$$

Then, each layer adaptively transforms the embedding using an additional 1×1 convolutional layer $w^{(l)}$:

$$z_{i,j,p}^{wp,(l)} = \sum_{k}^{C} z_{i,j,k}^{wp} w_{k,p}^{(l)} \tag{12}$$

The final condition embeddings $z^{wp,(l)}$ created in each layer are element-wisely multiplied with the output of the feed-forward network $z'^{(l)}$ before the residual connection in each layer:

$$\mathcal{L}^{(l)}(z^{(l)}) = z^{(l)} + z'^{(l)} \odot z^{wp,(l)} \tag{13}$$

where $z'^{(l)}$ refers to $W_2^{(l)} \sigma(W_1^{(l)} \tilde{\mathcal{K}}^{(l)}(z^{(l)}) + b_1^{(l)}) + b_2^{(l)}$ in eq (5). By injecting the condition into all layers through element-wise multiplication, we strongly regularize the spatial and structural information of the field patterns according to the varying wavelengths

4 Continuous-spectrum generalization capabilities of all compared models

Here, we provide the quantitative comparisons of the spectral generalization capabilities for all compared models. In the single-layer metalens (Figures S2 a and b), the proposed model consistently achieves low NMSE across the continuous broadband spectrum, including untrained wavelengths. In contrast, baseline models display notable error spikes at unseen wavelengths, demonstrating their limited ability to interpolate across the spectrum. Figures S2 c and d demonstrate the result of the color router simulation. The color router simulation presents a more challenging setting, exhibiting complex electromagnetic interaction due to high refractive index contrast. Under this configuration, baseline performance degrades substantially. However, our model continues to perform robustly across both the entire region and the design region. Finally, the freeform waveguide simulation features dense multilayer stacking and spatially unconstrained geometry (Figures S2 e and f). While UNet performed relatively well in earlier cases, its performance deteriorates significantly here, likely due to its localized receptive field and lack of spectral awareness. In contrast, the proposed model maintains low error and stability across the broadband spectrum.

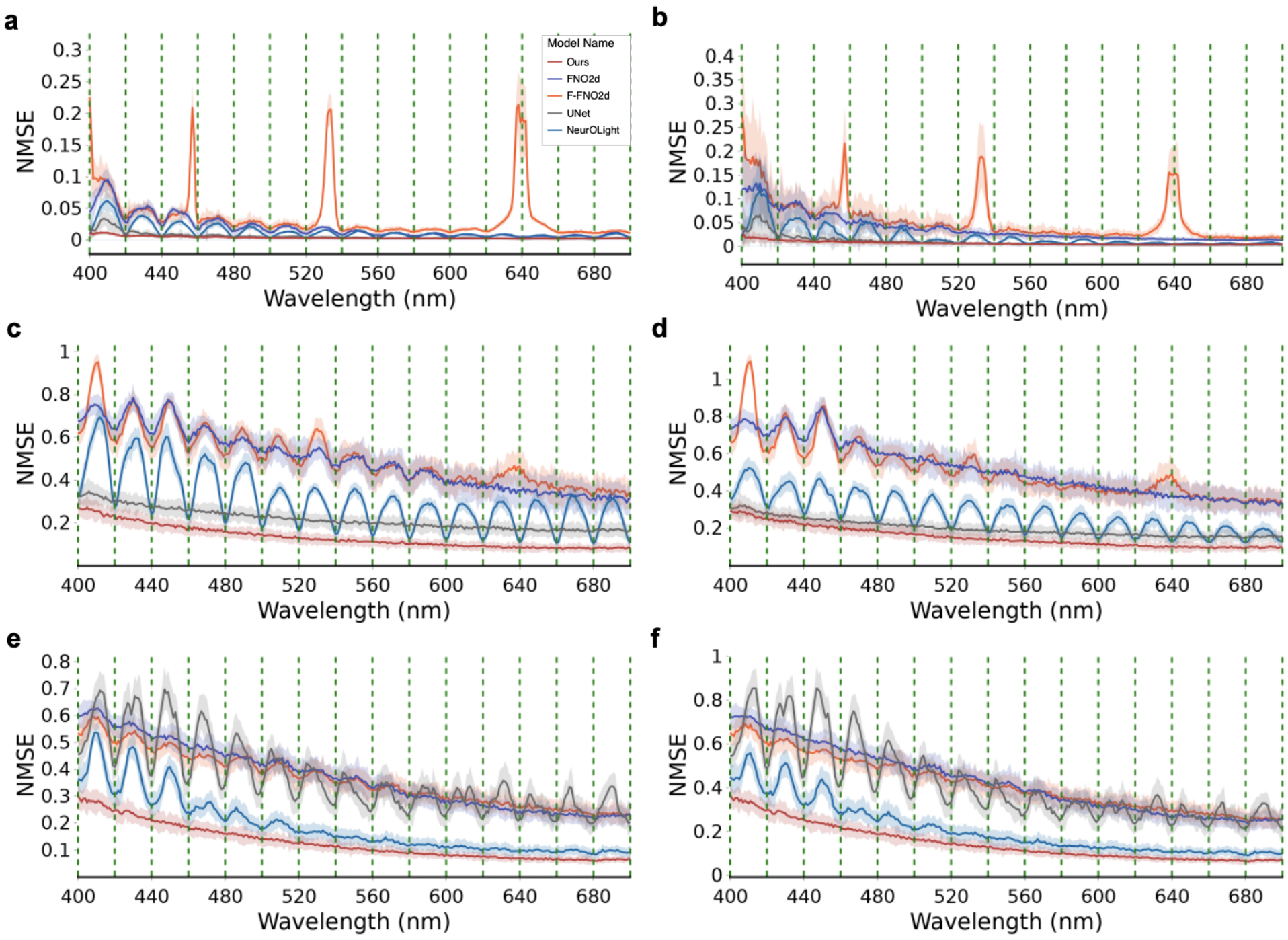

Fig. S2 Quantitative comparison of all compared models across the continuous and broadband spectrum. a-b. Results of the single-layer metalens case evaluated over (a) the entire domain and (b) the design region; c–d. Performance of the color router configuration evaluated over (c) the entire region and (d) the design region; e–f. Evaluation of the straight waveguide conducted over (e) the entire domain and (f) the design region. Shaded areas represent ±2 standard deviations for improved visibility, and green dashed lines indicate wavelengths used during training.

Consequently, these results demonstrate that our method reliably generalizes across untrained wavelengths and diverse structural conditions. The proposed model consistently outperforms conventional neural operators and CNN-based baselines by explicitly encoding wave-informed priors and enforcing spectral consistency.

5 Ablation Study

5.1 Hyperparameters of FGCS Operator

We assess the validity of the hyperparameters (groups and channels) and design choices (weight sharing and channel shuffling abbreviated by WS and CS) of the proposed FGCS operator by conducting an extensive ablation study, as shown in Table S1. We increase the number of groups in proportion to the growth of channels to consider a consistent number of parameters in all cases. Row 9 presents the FGCS operator used in our model. Rows 1-3, which do not utilize weight sharing and channel

Table S1 Ablation of design components of proposed FGCS operator. The ✓indicates the use of the design components. WS and CS indicate weight sharing and channel shuffling schemes.

WS	CS	Groups	Channels	Param (M)	Test loss		Design region test loss	
					Untrained	Trained	Untrained	Trained
		1	32	0.42	0.0040±0.0036	0.0029±0.0016	0.0059±0.0043	0.0055±0.0034
		2	48	0.56	0.0031±0.0027	0.0023±0.0013	0.0046±0.0034	0.0044±0.0029
		4	64	0.65	0.0028±0.0026	0.0019±0.0010	0.0039±0.0028	0.0038±0.0024
✓		2	48	0.37	0.0033±0.0026	0.0026±0.0014	0.0051±0.0033	0.0051±0.0031
✓		4	64	0.40	0.0029±0.0022	0.0023±0.0012	0.0045±0.0026	0.0045±0.0026
	✓	2	48	0.58	0.0028±0.0024	0.0021±0.0012	0.0044±0.0031	0.0042±0.0026
	✓	4	64	0.68	0.0024±0.0021	0.0017±0.0010	0.0037±0.0027	0.0035±0.0022
✓	✓	2	48	0.39	0.0030±0.0022	0.0024±0.0013	0.0049±0.0033	0.0047±0.0028
✓	✓	4	64	0.43	0.0024±0.0015	0.0021±0.0011	0.0042±0.0024	0.0042±0.0025
		8	128	1.90	0.0019±0.0020	0.0010±0.0006	0.0024±0.0022	0.0022±0.0015
✓		8	128	1.31	0.0014±0.0010	0.0011±0.0006	0.0022±0.0015	0.0022±0.0014
	✓	8	128	2.00	0.0017±0.0018	0.0009±0.0006	0.0022±0.0020	0.0019±0.0013
✓	✓	8	128	1.41	0.0013±0.001	0.0010±0.0006	0.0021±0.0015	0.0021±0.0014

shuffling in the Fourier domain, follow the same operational form as described in a previous study [15]. When only weight sharing is employed (rows 4-5), the prediction of fields for both unobserved and observed wavelengths during training demonstrates worse performance compared to that in the previous study due to a substantially small number of parameters.

In addition, comparing the previous method (rows 2-3) with the case where only channel shuffling is used (rows 6-7), the field prediction performances for both untrained and trained wavelengths are better. This suggests that employing the channel shuffling method with the grouping method significantly improves performance. Following the design configuration of the FGCS operator (rows 8-9), although the FGCS operator shows slightly lower prediction performance for trained wavelengths than when only a channel shuffling scheme is employed, it demonstrates competitive performance compared to the channel shuffling case, even with the much lower number of parameters.

Rows 10-13 present varying performances when increasing the model width. The performance improvement of the FGCS operator becomes increasingly significant with the addition of more channels. In particular, there is a substantial improvement when the number of groups and channels increases to 8 and 128, respectively. We believe that excessively sparse constructions of weight tensors and information compensating with channel shuffling may improve the generalization capacity of large models.

5.2 Permittivity in the Wave Prior

As shown in Table S2, we demonstrate performance improvements resulting from the exclusion of the ϵ_r term in the wave prior through an ablation study. To understand the underlying reason for this improvement, we analyze both the latent wave prior

Table S2 Ablation study for ϵ_r term in wave prior.

ϵ_r term	Test loss		Design region test loss	
	Untrained	Trained	Untrained	Trained
✓	0.0027±0.0019	0.0024±0.0013	0.0050±0.0033	0.0051±0.0031
	0.0024±0.0015	0.0021±0.0011	0.0042±0.0024	0.0042±0.0025

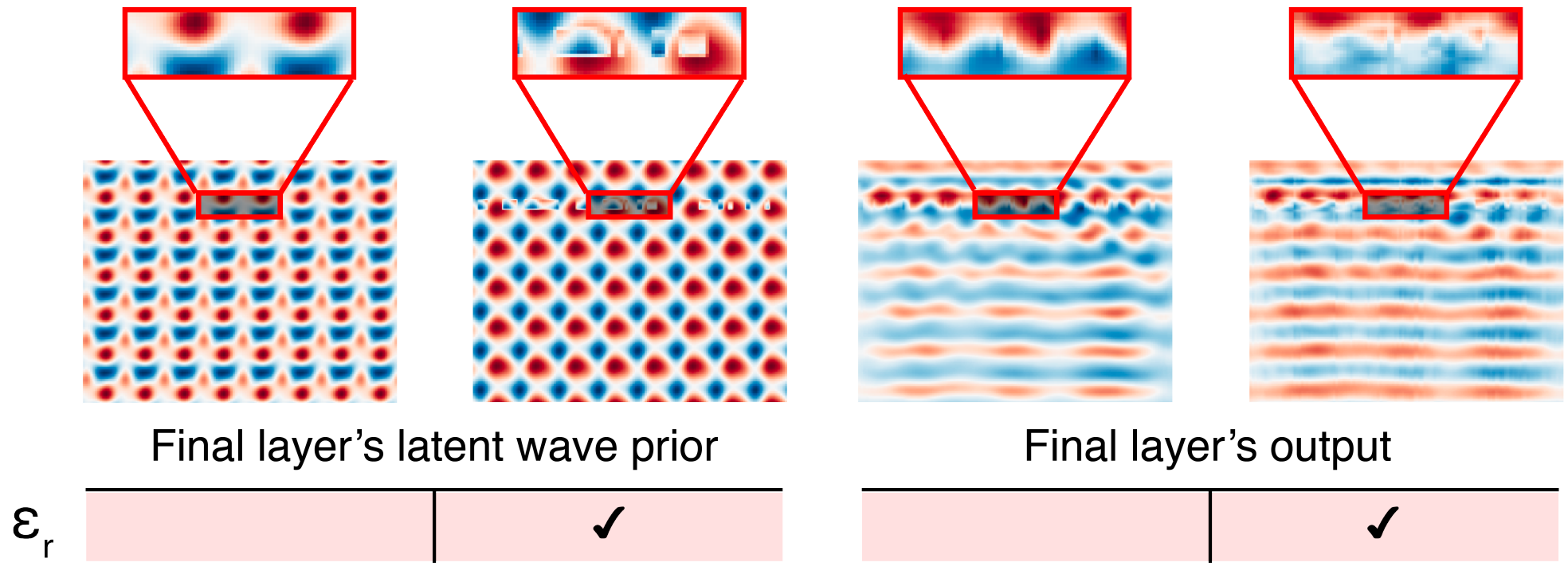

Fig. S3 Visualization of the features of condition information and outputs from the final layers of our model for a specific part of the entire region for 400nm wavelength data. (left) The latent wave prior in the final layer, excluding and including the ϵ_r term. (right) The output of the final layer without and with considering the ϵ_r term. The magnified sections are part of the region where structures exist.

and the output of the last layer in our model. Figure S3 (left) shows the latent wave prior before element-wise multiplication in the last layer of our model. Sharp artifacts emerge in regions containing structures, when the wave prior includes the ϵ_r term. This occurs because WIME utilizes shallow 1×1 convolutional layers, which maintain the spatial structure of the refined wave prior. Figure S3 (right) presents the final layer outputs of our model.

The result reveals that when the ϵ_r term is included in the wave prior, physically implausible artifacts appear throughout the entire region. Based on these findings, we conclude that incorporating the ϵ_r term in the wave prior introduces unintended bias, negatively impacting the model's field prediction accuracy and generalization capabilities.

5.3 Conditioning Methods

We conduct a comparative analysis of our conditioning method against two alternative approaches to assess its effectiveness.

Table S3 shows the results of the ablation study on different conditioning methods applied to our model. The conditioning methods considered for comparison include

Table S3 Ablation study of several conditioning methods.

Conditioning methods	Param (M)	Test loss		Design region test loss	
		Untrained	Trained	Untrained	Trained
Spectral parameter conditioning	1.09	0.1379±0.1049	0.0863±0.0280	0.1260±0.0464	0.1274±0.0460
Concatenating wave prior	0.33	0.0031±0.0029	0.0024±0.0013	0.0064±0.0075	0.0050±0.0028
WIME	0.43	0.0024±0.0015	0.0021±0.0011	0.0042±0.0024	0.0042±0.0025

Table S4 Comparisons of field prediction and interpolation with the simple concatenation conditioning and WIME for FNO and F-FNO.

Model	Param (M)	Test loss		Design region test loss	
		Untrained	Trained	Untrained	Trained
FNO	3.29	0.0168±0.0162	0.0093±0.0083	0.0303±0.0260	0.0273±0.0229
FNO-WIME	3.30	0.0122±0.0124	0.0105±0.0092	0.0290±0.0231	0.0301±0.0251
F-FNO	1.89	0.0379±0.0443	0.0254±0.0561	0.0474±0.0457	0.0389±0.0643
F-FNO-WIME	1.92	0.0086±0.0058	0.0081±0.0056	0.0168±0.0127	0.0172±0.0129

spectral parameter conditioning [16, 17] and the concatenating wave prior [8]. A detailed explanation on the conditioning methods is in the Appendix Section D.

The spectral parameter conditioning method fails to generalize across the broadband spectrum and exhibits limited accuracy in predicting electromagnetic fields within the design region, even at wavelengths included during training (Table S3, row 1). This suggests that it not only lacks generalization capability but also struggles to capture the complex electromagnetic phenomena occurring in the design region. In contrast, incorporating wave priors significantly improves the model's ability to learn intricate field patterns and enhances generalization to untrained wavelengths across the spectrum (see Supplementary Table S3, rows 2–3). Furthermore, WIME outperforms simple wave prior concatenation in both trained and untrained wavelength settings. This result demonstrates that our method effectively incorporates condition embeddings that closely align with the spatial and structural features of the fields, leading to more accurate and robust predictions across a continuous range of wavelengths.

Furthermore, we conducted an additional comparative analysis between WIME and other conditioning methods using various models. "FNO" and "F-FNO" refer to methods that incorporate the wave prior by concatenating it with the input data. A detailed description of these conditioning methods is provided in the Detailed Conditioning Methods section.

As shown in Table S4, integrating WIME with FNO enhances prediction performance for unseen wavelengths during training. In particular, applying WIME to F-FNO leads to substantial improvements in field prediction for both trained and untrained wavelengths. The improvement stems from WIME's ability to effectively

incorporate condition information, thereby enhancing the performance of F-FNO. Moreover, WIME successfully addresses the information loss caused by the factorization of the Fourier transform over the problem dimensions. These findings confirm that our conditioning method significantly enhances generalization capability, particularly in scenarios where the model is trained on a limited and discrete set of observed wavelengths.

6 Detailed Conditioning Methods

6.1 Spectral parameter conditioning

The spectral parameter conditioning method transforms the scalar wavelength into a vector via sinusoidal embedding [12]. It then employs a two-layer feed-forward network to project the embeddings onto a higher-dimensional space of 4 $\times$ hidden channels. The embeddings are mapped to the Fourier space using the module named FreqLinear module described in [16]. Finally, the embeddings in the Fourier space are mode-wise multiplied with the input of the Fourier integral kernel to perform parameter conditioning in the spectral domain.

6.2 Concatenating wave prior

The concatenating wave prior injects the condition information by simply concatenating the given refined wave prior to the input data.

7 Detailed Model Architectures

7.1 Unet

We use a 4-level modern convolutional Unet architecture with an initial channel size of 16. Each level consists of two residual blocks with GELU activation function and Group Normalization. We concatenate the refined wave prior with the input for the conditioning method.

7.2 FNO

We set the number of Fourier layers and the number of channels in each Fourier layer to 5 and 32, respectively. The frequency modes for the z and x axes are set to [32, 10]. In addition, the lifting operator is a linear operator, and the projection operator is a 2-layer feed-forward network with GELU activation and a dimension of 128. We concatenate the refined wave prior with the input to inject condition information.

7.3 F-FNO

We employ 12 Fourier layers, each consisting of 64 channels. The frequency modes for the z and x axes are set to [50, 60]. The lifting operator is a linear operator, and the final projection operator is a 2-layer feed-forward network with GELU activation function and a dimension of 256. To incorporate condition information, we concatenate the refined wave prior with the input.

7.4 NeurOLight

We use 12 Fourier layers with 64 channels each. The frequency modes for the z and x axes are set to [50, 60]. The channel expansion factor for the convolutional modules in the NeurOLight layers is configured to be 2. To inject condition information, we concatenate the wave prior with the input. During training, we implement stochastic network depth with a rate of 0.1 to the residual NeurOLight layers to alleviate overfitting.

7.5 Ours

We use 12 layers with 64 channels each and specify the number of groups as 4. The frequency modes for the z and x axes are configured as [50, 60]. We implement a channel expansion scheme with a factor of 2 for the 2-layer feed-forward network in the layers, inspired by [8, 18]. The lifting operator is linear, and the final projection operator is a 2-layer feed-forward network with GELU activation and a dimension of 256.

8 Simulation Setup

In our study, we utilize an open-source FDFD simulation tool Ceviche [9] to generate electromagnetic field data for photonic structures. All simulations employ a uniform grid resolution of 40 points per micrometer and include a 1-μm-thick perfectly matched layer at the boundaries. For the single-layer metalens, we simulate lens geometry over a 5.2 μm $\times$ 6.57 μm domain using SiO_2 (refractive index 1.46) and air in 25 nm grid patches. The multi-layer spectrum splitter is modeled on a 5.2 μm $\times$ 9.05 μm domain comprising five layers, each composed of high-permittivity (4.0) and low-permittivity (2.13) materials, and is 700 nm thick. The freeform straight waveguide is simulated on a 5.2 μm $\times$ 8.85 μm domain in which polymer ($\epsilon_r = 2.25$) and air regions are arranged in 25 nm–scale patches within an entirely unconstrained design region. In each case, we generate the electric field distributions under normally incident plane-wave illumination for wavelengths spanning 400 nm to 700 nm.

References

[1] Jiang, J., Chen, M., Fan, J.A.: Deep neural networks for the evaluation and design of photonic devices. Nature Reviews Materials **6**(8), 679–700 (2021)

[2] Trivedi, R., Su, L., Lu, J., Schubert, M.F., Vuckovic, J.: Data-driven acceleration of photonic simulations. Scientific reports **9**(1), 19728 (2019)

[3] An, S., Zheng, B., Shalaginov, M.Y., Tang, H., Li, H., Zhou, L., Ding, J., Agarwal, A.M., Rivero-Baleine, C., Kang, M., *et al.*: Deep learning modeling approach for metasurfaces with high degrees of freedom. Optics Express **28**(21), 31932–31942 (2020)

[4] Chen, M., Lupoiu, R., Mao, C., Huang, D.-H., Jiang, J., Lalanne, P., Fan, J.A.: High speed simulation and freeform optimization of nanophotonic devices with physics-augmented deep learning. ACS Photonics **9**(9), 3110–3123 (2022)

[5] Azizzadenesheli, K., Kovachki, N., Li, Z., Liu-Schiaffini, M., Kossaifi, J., Anandkumar, A.: Neural operators for accelerating scientific simulations and design. Nature Reviews Physics, 1–9 (2024)

[6] Li, Z., Kovachki, N., Azizzadenesheli, K., Liu, B., Bhattacharya, K., Stuart, A., Anandkumar, A.: Fourier neural operator for parametric partial differential equations. arXiv preprint arXiv:2010.08895 (2020)

[7] Tran, A., Mathews, A., Xie, L., Ong, C.S.: Factorized fourier neural operators. arXiv preprint arXiv:2111.13802 (2021)

[8] Gu, J., Gao, Z., Feng, C., Zhu, H., Chen, R., Boning, D., Pan, D.: Neurolight: A physics-agnostic neural operator enabling parametric photonic device simulation. Advances in Neural Information Processing Systems **35**, 14623–14636 (2022)

[9] Hughes, T.W., Williamson, I.A., Minkov, M., Fan, S.: Forward-mode differentiation of maxwell's equations. ACS Photonics **6**(11), 3010–3016 (2019)

[10] Hughes, T.W., Minkov, M., Williamson, I.A., Fan, S.: Adjoint method and inverse design for nonlinear nanophotonic devices. ACS Photonics **5**(12), 4781–4787 (2018)

[11] Kovachki, N., Li, Z., Liu, B., Azizzadenesheli, K., Bhattacharya, K., Stuart, A., Anandkumar, A.: Neural operator: Learning maps between function spaces with applications to pdes. Journal of Machine Learning Research **24**(89), 1–97 (2023)

[12] Vaswani, A., Shazeer, N., Parmar, N., Uszkoreit, J., Jones, L., Gomez, A.N., Kaiser, Ł., Polosukhin, I.: Attention is all you need. Advances in neural information processing systems **30** (2017)

[13] Xie, S., Girshick, R., Dollár, P., Tu, Z., He, K.: Aggregated residual transformations for deep neural networks. In: Proceedings of the IEEE Conference on Computer Vision and Pattern Recognition, pp. 1492–1500 (2017)

[14] Guibas, J., Mardani, M., Li, Z., Tao, A., Anandkumar, A., Catanzaro, B.: Adaptive fourier neural operators: Efficient token mixers for transformers. arXiv preprint arXiv:2111.13587 (2021)

[15] Kim, M., Park, J., Shin, J.: Efficient fourier neural operators by group convolution and channel shuffling. In: ICLR 2024 Workshop on AI4DifferentialEquations In Science (2024). https://openreview.net/forum?id=TygIZCofE3

[16] Gupta, J.K., Brandstetter, J.: Towards multi-spatiotemporal-scale generalized

pde modeling. arXiv preprint arXiv:2209.15616 (2022)

[17] Mao, C., Lupoiu, R., Dai, T., Chen, M., Fan, J.A.: Towards general neural surrogate solvers with specialized neural accelerators. arXiv preprint arXiv:2405.02351 (2024)

[18] Xie, E., Wang, W., Yu, Z., Anandkumar, A., Alvarez, J.M., Luo, P.: Segformer: Simple and efficient design for semantic segmentation with transformers. Advances in neural information processing systems **34**, 12077–12090 (2021)
